# The Medical Jurisprudence of Insanity

**Published:** 1840-07

**Authors:** 


Art. VI.

1. *The Medical Jurisprudence of Insanity.* By J. M. Pagan, m.d., Lecturer on Medical Jurisprudence, &c.—*London and Glasgow*, 1840. 8vo, pp. 327.

2. *A Treatise on the Medical Jurisprudence of Insanity.* By J. Ray, m.d. *With an Introductory Essay by* D. Spillan, m.d.—*London*, 1839. 8vo, pp. 430.

3. *Du Suicide, de l'Aliénation Mentale, et des Crimes contre les Personnes.* Par J. B. Cazauvieilh, Docteur en Médecine.—*Paris*, 1840. pp. 325.

*On Suicide, Insanity, and Crimes against the Person.* By J. B. Cazauvieilh.—*Paris*, 1840.

4. *A Letter to the Right Honorable the Lord Chancellor, on the present state of the Law of Lunacy; with Suggestions for its Amendment.* By a Barrister of the Inner Temple.—*London*, 1838. pp. 16.

5. *De la Folie, considérée dans ses Rapports avec les Questions Medico-judiciaires.* Par C. C. H. Marc, Premier Médecin du Roi, &c.—*Paris*, 1840. T. II. pp. 560-738.

*Insanity considered in its Medico-legal relations.* By C. C. H. Marc, Chief Physician to the King, &c.—*Paris*, 1840. Two Vols.

In the Thirteenth and Seventeenth Numbers of this Journal, we have devoted articles of some length to the subject of Insanity. In the first place we have presented our readers with the opinions of the most experienced modern writers on the causes, varieties, and treatment of mental alienation; and secondly, we have dwelt particularly on the rules for the organization and management of lunatic asylums. It is now our purpose to call the attention of the profession to those points connected with the subject of insanity, which the length of our former articles did not allow us to notice. The medico-legal questions which affect the insane are admitted by all to be of paramount importance; and it is much to be regretted that in this country so little attention has been hitherto paid by members of the medical and legal professions to questions of such vital interest. The confinement of persons on groundless allegations of insanity—the premature liberation of lunatics supposed to be cured—the setting aside of wills and deeds executed by the insane—the annulling of the marriage contract—and, lastly, the execution of persons for crimes committed while in a state of insanity, are surely circumstances which must forcibly fix the attention even of the most indolent practitioner, especially when he considers that in each case he himself may become the instrument of the improper administration of the law.

Three works on the Medical Jurisprudence of Insanity have recently appeared: one by Dr. Ray, an American physician; one by Dr. Pagan, of Glasgow; and a third by M. Marc, of Paris, whose premature death, we regret to say, immediately followed the completion of his work. We shall commence with the work of the Scottish author.

Dr. Pagan first considers "*unsoundness of mind,*" as it is defined by lawyers, and he then passes to the medical signification of the term "insanity." After a few remarks upon the causes of mental derangement, he gives a short summary of each of its forms, adhering to the divisions proposed by Pinel and Esquirol, and now generally followed by the profession. The medico-legal relations of each of these divisions are distinctly and successively considered. Thus following the chapter on Mania, we have an exposition of the circumstances which may justify restraint—the facts connected with the execution of wills and deeds—with the plea of irresponsibility for crimes committed during this state—the means of detecting concealed and feigned mania, &c. This plan necessarily involves some repetition, since each question is separately reconsidered for each form of insanity; but we do not regard this as a fault in a volume expressly intended for practical reference and assistance. We perfectly agree with the remark of the author in his preface, that this is the arrangement by which correct notions of the various forms of unsoundness of mind, and of the medico-legal consequences that result therefrom, can be most easily obtained.

It is well known that no consistent legal definition of what constitutes unsoundness of mind ("*non compos mentis*") has yet been given. No two Chancellors have agreed upon the exact grounds on which a writ "de lunatico inquirendo" should be issued. Lord Hardwicke held that there must be a depravity of reason or a want of it; and he drew, to us, an unintelligible distinction between "infirmitas mentis" and "insanitas mentis." The former case involving *weakness* of mind, with incapacity on the part of a person for managing himself or his affairs, was not, in his view, a subject for enquiry. Lord Eldon dissented from this doctrine, and issued a commission where there was imbecility, not strictly amounting to insanity, but calling for as much protection from the law as actual insanity. In looking at the conflicting decisions on this point, all we can say is that unsoundness of mind involves a morbid or at least a defective condition of intellect, with an incompetency on the part of the person to manage his affairs. Neither condition will suffice without the other; for the intellect may be in a morbid state, and yet there may be no incompetency; or the incompetency may depend on bodily infirmity or a want of education, and may be purely relative—that is to say, vary with the station of the individual in society.

We fully agree with Dr. Pagan that all the law-definitions of insanity teem with error; that they involve absurdities long since exploded; and that they are very far behind the present state of psychological knowledge. He condemns Lord Coke's definition of "*non compos mentis*" as erroneous and imperfect; indeed, we may say that it has but little more relation to insanity than to witchcraft; and yet we find it sometimes followed as authority in law proceedings. The iron rule of precedent thus binds down the judgment; and often prevents the law from receiving that light from the advance of modern science, of which, in reference to the questions we are now considering, it stands so much in need.

From a review of the arguments of counsel in many cases of contested insanity, we might be almost led to suppose that the arbitrary dicta of great law-authorities, who lived 200 years ago, were of more importance in settling these questions than the knowledge derived from a careful observation of disorders of the mind. Thus in one case it was contended "that the law only recognizes two kinds of insanity—lunacy and idiocy, and although lunacy has been of late described in other words as '*non compos mentis*,' yet the words only were changed, not the law." When we consider that the legal definitions of lunacy and idiocy are, in a medical point of view, totally inapplicable to those states, and that some of our judges are extremely jealous of admitting any others, we shall be prepared to understand the kind of embarrassment under which a medical witness may labour, who is called to give evidence in a particular case. The person must be proved either lunatic or idiot, and that not in a medical but in a legal sense. This is a reversal of the old French axiom: "Quand les hommes disent que telle chose est, et la nature dit que telle chose n'est pas, il faut en croire la nature." How is a medical witness to conduct himself? He may not be able to swear that in his opinion the party comes up to the strict *legal standard* of lunacy or idiocy; and yet he may not have the smallest doubt of his mental incapacity. His rule of conduct should be to examine the case repeatedly; and having formed an opinion of the state of mind of the party, he should give his evidence accordingly, "disregarding all descriptions, all standards, all legal definitions whatever." From the excellent decisions which have been given of late years in our ecclesiastical courts, in cases of contested wills, there is great reason to believe that a wider and more comprehensive view of this subject is developing itself among our law authorities: and that in future, questions of this kind will be decided less by antiquated precedents than by sound medical opinions.

By a late Act of Parliament it is settled that the term lunatic shall extend to any idiot or person of unsound mind, or *incapable of managing his affairs*. If, as it is said, the last term is to be taken as a legislative exposition of the first, then the subject will become henceforth much simplified. We shall hear no more of casuistical distinctions made between weakness and unsoundness of mind; the simple test for legal interference will be an incapacity on the part of a person, from decided *mental infirmity*, to manage his own affairs.

It is not our intention to follow the author into the medical definitions of insanity; we agree that they are imperfect, and that it would be difficult to find one which includes all who are insane, and excludes all who are not. The reason for this is obvious in the varying characters and in the imperceptible gradations of disordered intellect observed in insanity. The following description comprises the class of cases which offers the greatest difficulties to the medical jurist:

"The senses perform their functions incorrectly; the faculties of attention, comparison, and association, &c. are likewise in a diseased state, and the person of course is insane. But it is not to be presumed that in every case of insanity all the intellectual faculties are in such a state of disease as to be incapable of performing, in some degree, their usual office. There are cases of general insanity in which the function of no one of the faculties of the mind can be observed to be performed even in the most imperfect manner. There are other instances in which all the faculties seem to be annihilated; no mental operation, so far as we can judge, is capable of being performed. But it far more frequently happens that the mental and moral powers are partially diseased or partially destroyed. Now the degree of this state of disease or decay is very various, and the kind of it is equally so; these varieties most probably being dependent upon the extent of disease in certain faculties and the integrity of others—the combination of faculties affected—how far the senses perform their functions incorrectly—in what manner and to what degree. For example, in reference to the sense of sight, whether objects appear larger or smaller, or of what colour, at what distance, and so forth. The whole of the faculties of the mind are liable to become diseased; they may each or all of them be affected, and they may be diseased in various degrees and in various combinations. The combination may unite disease of the intellectual with the moral faculties; and, I believe, it is no uncommon thing to find cases in which, along with depravation of some of the faculties of the mind, there is exaltation and increased power of some other, as, for example, the imagination." (*Pagan*, p. 20.)

It is important for a medical jurist to bear in mind that insanity does not exclusively consist in disease of the faculty of attention, in errors of perception and comparison, exaltation of the imagination, and defective judgment. Something besides the intellectual faculties must be taken into consideration, as, for example, those important powers by which men are influenced to good or evil—the moral faculties, the passions, the affections. These exercise the most important influence over the individual, not merely as a moral, but as an intellectual being. "Disease of the moral faculties may exist when it is impossible to discover any intellectual disorder." (p. 23.) While the author does not deny that a man may be insane from a disease of the moral as well as of the intellectual faculties, he thinks that the former most frequently includes or induces disease of the intellectual powers likewise. The condition of the moral faculties, therefore, requires as close an observation as that of the intellectual.

The following remarks are worthy of attention:

"The insane are for the most part totally unconscious of their condition. They believe themselves to be in the enjoyment of perfect health; they are satisfied that their feelings are real, and that their thoughts are true; and any attempt to reason them out of their belief in their sanity would be just as idle as to endeavour to persuade a man of sound mind that his feelings and thoughts were unreal and untrue. The principle of belief in both is the same. This fact is of importance in reference to medical jurisprudence. There are exceptions to this rule, however. Some are well aware that their intellectual faculties are in a disordered state: and it is a symptom almost characteristic of diseased volition for the patient to be quite conscious of his unfortunate malady." (*Pagan*, p. 26.)

Georget remarked that the insane either forgot altogether the circumstances which preceded the attack, or they were continually recalled and became the cause or the pretext of all their intellectual disorder. He also thought that occurrences which take place during the disease are minutely remembered after recovery. This latter opinion is not universally correct; but that the power of memory may exist to a remarkable extent, even where it is supposed to be absent, is clearly shown by numerous facts.

We shall pass over the *causes* of insanity, as these have been amply discussed. The characters of mania present nothing new. The diagnosis of mania is of considerable importance, and in a medico-legal view it is especially necessary to distinguish between it and delirium. There is, it is well known, a close resemblance between the delirium which proceeds from cerebral disease and the acute form of mania. They are both attended with the same intellectual and moral aberrations, and the same degree of excitement, amounting often to outrage and fury.

"But the mode of aggression is very different. The alienation of the mental faculties is the first symptom which presents itself in an attack of mania, while delirium succeeds to other symptoms of physical disorder: delirium is for the most part attended with febrile excitement to a greater extent than is found in mania. Delirium and mania differ in their mode of aggression, in their progress, and termination—delirium being altogether an accessory of the disease of which it is a symptom; it exists as long as the disease on which it depends, and no longer. In short, the previous existence of physical disease, the mode of aggression, the progress and termination, sufficiently distinguish delirium from mania." (*Pagan*, p. 69.)

In delirium tremens, consequent on the abuse of intoxicating liquors, the attack generally commences with tremors of the hands, great restlessness and long-continued watching. Illusions of various kinds, and often having reference to past occupations, with hallucinations succeed. Patients in this state are frequently violent, and are especially prone to the commission of suicide.

Mistakes are said to have occurred where persons labouring under delirium have been ordered into confinement as maniacs—thus seriously compromising the reputation of a practitioner. With the use of ordinary care, the diagnosis is not likely to be difficult.

On the subject of personal restraint in mania the author prudently adopts a *mezzo termine*. The object of restraint, he says, is twofold: to prevent the injurious consequences which might result to the maniac himself or to others from his remaining at large, and to contribute as much as possible to the cure of the disease. The first is undoubtedly the more important object, by which a medical jurist must be guided; the second is rather a question for the physician, and it is one upon which opinions are considerably divided. The abuses to which the system of restraint formerly led have produced a complete revulsion in medical opinion. The author appears to us to discuss the point with candour and good feeling.

"Whenever the patient has given us the slightest reason to fear that his own safety, or that of his associates or friends, is in danger, and if his change of character threatens injurious consequences to his own or his family's interests—if he be recklessly squandering away his means—if he be irritable or outrageous, and threatening personal violence to any one, then we are quite justified in having him placed under such restraint as will not only most effectually prevent any accidental injury to himself or others, but will be conducive to his cure. The patient cannot sustain any injury by our conduct being thus regulated. It is certainly no light matter to interfere with the personal liberty of any one, and the law is very properly highly jealous on this point, even in the case of insanity; but it is better that a man should suffer the imposition of slight restraint than that he should be injured either in his person or his means, or in those of his family or friends. Change of character is not insanity; but it may be evidence of insanity, and it is a most unusual thing for men all at once to assume a character absolutely foreign to their natures, independently of incipient unsoundness of mind." (*Pagan*, p. 75.)

Violence of temper, haughtiness, and eccentricity must not be mistaken for incipient insanity. We are not merely to compare one man with his fellows; for in this case eccentricity or fits of violent passion might be pronounced evidence of insanity. We must find out the change of character by enquiring whether the individual is different at one time to what he has always been—whether his habits and feelings have suddenly undergone any unusual change. A violent-tempered man may have fits of greater violence than common; but still this is his natural character, and must not be set down as indicative of insanity.

We seldom find questions raised in our law-courts as to the validity of wills and deeds executed by persons in a state of mania; but it is by no means unusual in the incipient stage that this should be brought forward as a plea of irresponsibility for crimes. Persons thus affected seldom commit murder, except when impelled to it by accidental circumstances, such as the attempt to enforce restraint, or to interfere with habits depending on their hallucinations. These are cases of difficulty. Sometimes the crime itself may have actually been the exciting cause of the disease. A person labouring under insanity is not liable to punishment, even for a crime committed previous to the attack; but the same exception holds with one labouring under bodily disease. In either case they would be liable to punishment on recovery; while if the crime were committed during an attack of insanity, the individual would be wholly exempt from punishment even after recovery. It is a fact worthy of notice that, while the commission of a crime has sometimes suddenly led to the access of insanity in a previously sane person, the sight of the murdered victim has often suddenly restored the insane perpetrator of a crime to reason. In the first case we should be apt to suppose that the insanity was feigned; in the second that it had not existed during the commission of the crime; and in either case, unless we reflected deeply on the circumstances, we might lead to the unjust interposition of the law. In the following is a case where an attack of mania occurred so speedily after the commission of a criminal act as naturally to have led to the suspicion that the disease was feigned.

"Two men were committed to prison on a charge of theft; and the officers who had them in charge, fearing that their prisoners might make an attempt to escape, at a lonely part of the road over which they had to travel, called upon a man by the way, and requested, as a favour, that he would assist them in conveying the prisoners to gaol. The poor man was sitting at home, quietly pursuing his trade of a shoemaker, and upon the representation of the officers as to their fears of an escape being attempted, he agreed to accompany them, and to take a gun with him for greater security. As had been anticipated, one of the prisoners leaped from the cart and ran off. The officers called to their assistant to fire, and he, thinking himself warranted to do so, under the circumstances and by their authority, fired, and wounded the man severely in the back and loins. He was himself immediately committed to gaol." (*Pagan*, p. 82.)

The whole of the circumstances had such an effect upon him, that he became violently maniacal. When hardly recovered, he was tried for the offence, convicted, and sentenced to six months' imprisonment. Owing to a proper representation on the state of the man's mind being made to the court, the sentence was not carried into effect.

In determining whether a *lucid interval* exists or not, "it is a useful criterion, to find out whether the person remembers what passed during the active stage of his disease. A distinct and accurate recollection of what passed during the active form of mania, is a favorable circumstance, as it is not, in the majority of cases, until the mental powers are restored to a state of comparative good health, that this is found to be the case." (p. 86.) The art with which a patient will endeavour to avoid all questions bearing on the subject of his delusion is well known; and the self-command which they are capable of displaying is illustrated by many recorded cases. A firm resolution on the part of a patient will sometimes baffle every endeavour to make him betray the nature of his delusion; and, unless some clue to this be previously obtained by the examiner, he may leave the patient with the impression that he is perfectly sane. Dr. Pagan recommends that we should first try to gain the patient's confidence, by not contradicting him, but rather seeming to agree with him. Should this mode not be successful, " it may be proper to contradict him, and even irritate him so as to put him into a passion, when his caution will in all probability be got the better of." We doubt the propriety of following this last piece of advice, since some men whose only infirmity may be that of having naturally a violent temper might thus incur the risk of being locked up as insane. Dr. Pagan must be aware, from the rules which he has already laid down, that when a man is overcome by passion purposely excited, it is not exactly the time for a medical practitioner or a jury to pronounce an opinion on his sanity or insanity. We do not know any greater absurdity than that of putting abstruse metaphysical questions, as on the power and attributes of a Supreme Being, to one who never heard of metaphysics; or of asking difficult arithmetical questions of one who perhaps never advanced so far as the rule of three;—and yet it would be easy to bring forward numerous cases in which the insanity of parties has been actually tested by criteria so loose as these. The truth is, we can never compare the mind of one man with the mind of another without taking into consideration the various degrees of intellectual power with which men are naturally gifted, as well as the influence of their respective social positions in the development of their faculties. We must not expect more from a man's mind than it has shown itself capable of receiving, or in the words of our author: " We can only draw correct inferences as to the soundness of mind of any given person by comparing his present opinions with what it is certain that he must once have known." (p. 93.) That practitioner, therefore, is only fitted to conduct an examination of a supposed lunatic, whether the question relate to the commencement of insanity, the occurrence of a lucid interval, or to alleged recovery, who has made himself thoroughly acquainted with the past habits of the patient and the opportunities which he may have enjoyed of cultivating his mind.

The author does not notice the excellent rule suggested by Dr. Conolly, for testing the state of the mind in these cases, namely, that of inducing the patient to express his thoughts in writing, as in a letter addressed to his physician or to some confidential friend. We are satisfied that this would often answer in developing the existence of a delusion, where an examination would wholly fail. The current of thoughts would here be uninfluenced by the suspicion that the act of writing was to test the state of mind; and as no man can long write in a connected manner who does not think collectedly, so we should soon have ample evidence whether a delusion existed or not. Dr. Pagan furnishes, unknowingly, a remarkable instance of the efficacy of this plan at p. 19, where he describes the case of a lady of unsound mind who wrote a letter to a friend, in which was a quotation from scripture. She gave a correct reference to the part of the Bible where the passage was to be found—thus, Philippians, iii. 7, and immediately added, "These islands lie in latitude north,—and longitude——;" most probably referring to the geographical position of the Philippine islands. Here was undoubtedly a defect in the faculty of association; and as this defect, coupled with an imperfect state of the powers of attention and comparison, exists to a greater or less extent in all cases of insanity, so we may expect that this method will often enable a practitioner to form a satisfactory opinion of the case.

When mania is *feigned*,—and the remark holds good in reference to the simulation of other diseases,—the part is generally over-acted. An impostor always thinks that he cannot crowd a sufficient number of symptoms into his disease; and this over-acting of the assumed part frequently affords the very best means of detecting the deceit. It is important to remember,

"As a valuable distinction between mania, real and feigned, that the really insane person rarely acknowledges his belief in his melancholy situation: on the contrary, he generally fancies himself to be perfectly sane, and prides himself on the correctness of his judgment. In the feigned case, we may have no attempt made directly to persuade us that he believes himself to be insane, but the impostor will make no endeavour to convince us that he is of sound mind. Tell an insane patient that he is mad, he instantly contradicts you: while if you express your belief in the hearing of an impostor that he is really insane, you will be met by no contradiction; so far from this, he will most probably endeavour to illustrate the truth of your remark by conduct which he thinks will be deemed conclusive evidence of his insanity." (*Pagan*, p. 97.)

Dr. Pagan thinks that persons who are habitual drunkards should be placed under some legal restraint like lunatics. The justification for this would be the protection of the patient and his friends from the effects of his misconduct, and the promotion of his cure. Some German writers have treated of confirmed drunkenness as a variety of cerebral disorder, under the name of dipso-mania. The reasons assigned for restraint are good; and when we consider that habitual drunkenness, by weakening the powers of the mind, is allowed as an excuse for crime, it becomes the more necessary that the legislature should cautiously interfere; since otherwise it is laying down the rule, that a person whose state of mind is such as to render him irresponsible for the most heinous crime may be allowed with impunity to bring ruin upon himself and family.

Persons labouring under delirium tremens are considered incompetent by our law to execute a deed or devise property by will; but the incompetency only exists during the attack, for should the mind clear up, as in such cases it very often does immediately before death, then the individual may have so perfect a restoration of his faculties as to enable him to dispose of his property by will. These lucid intervals have been observed to occur more particularly in those cases where, after the subsidence of delirium the patient has sunk in consequence of some other physical disease. This fact is of importance to the medical jurist.

"Delirium tremens does not always immediately follow the excessive use of intoxicating drinks:—in many, perhaps in most cases, it does not make its appearance till the person, who has addicted himself to this vice, has abstained from their use for a certain length of time." (*Pagan*, p. 118.)

The law does not hold persons responsible for crimes committed during an attack of delirium tremens, or delirium depending on cerebral disease. Acquittals have even taken place on charges of murder where there was deliberation and an apparent motive for the act. We agree with the author that, morally speaking, there can be no difference between the criminality of acts committed under the influence of intoxication from indulgence to excess in intoxicating liquors, as an *immediate* consequence, and that of acts committed during delirium from the long-continued abuse of ardent spirits, as a *remote* consequence; but still the law admits a difference.

A considerable portion of Dr. Pagan's work is devoted to the subject of monomania under its various forms, as shown in a propensity to homicide, suicide, incendiarism, theft, demonomania, &c.; but we shall pass to the medico-legal applications of these varieties of insanity, merely observing that the symptoms and rules for diagnosis are amply and satisfactorily detailed. The first question relates to the restraint of patients affected with monomania; and here a nicer discrimination is required on the part of a practitioner than in a case of general insanity.

The mere existence of a delusion in a person is not of itself sufficient to justify the application of personal restraint. If there be an instinctive impulse to murder, or burn, or steal, or destroy everything which comes within his reach, there can be no doubt of the propriety of confining him until these tendencies have disappeared; and this becomes the more easy, since in such cases the patients themselves are conscious of and lament their infirmity. (p. 187.) Confinement should not be continued longer than is absolutely necessary for the object for which it has been imposed. Here again, we must guard against the danger of releasing a patient too soon.

Monomania is seldom *feigned*, from the difficulty, even to the most accomplished impostor, of acting up to the assumed character. Insanity is never, or but rarely, feigned for the purpose of avoiding the *suspicion* of guilt;—for this would be the most certain means of inviting suspicion. The imposition is only attempted when detection becomes inevitable.

The validity of deeds executed by persons affected with monomania, often becomes a subject of dispute. The practice of the law here indicates that the mere existence of a delusion in the person, does not necessarily vitiate the deed, unless the delusion form the groundwork of it, or unless the most decisive evidence be given that at the time of executing it the testator's mind was influenced by the delusion. Strong evidence is always derivable from the act itself, especially where the testator has drawn it up of his own accord. But the deed may be manifestly unjust to his surviving relatives, and it may display some of the extraordinary opinions of the individual, yet it will not necessarily be void, unless the testamentary dispositions clearly indicate that they have been framed under a delusion. Some injustice may possibly be done by the rigorous adoption of this principle; since delusion may enter into a man's act, without our being always able to discover it.

The evidence in these cases sometimes only amounts to proof of eccentricity on the part of the testator, or in the deed itself; but a clear distinction must be here drawn. The will of an eccentric man is such as might always have been expected from him: the will of one labouring under delusion is different from that which he would have made in an unaffected state; the instrument is wholly different from what it would once have been.

In the case of a Mr. Stott, a medical electrician, whose will was disputed by his daughter on the ground of insanity, it was proved that this gentleman fancied that he could deliver pregnant women by means of electricity; but the deed was set aside, not so much on the ground of this absurdity, as of the violent and unnatural treatment to which he had subjected his daughter. It appeared that he had taken, as we now and then find in monomaniacs, a most unaccountable and causeless dislike to this girl from her earliest infancy.

Wills are sometimes contested more on the ground of eccentricity than on that of insane delusion, but the cases are different; and while in the one the validity is denied, in the other it is allowed. Dr. Pagan does not seem to have met with a remarkable case of this kind, decided in 1838, in our Ecclesiastical Courts,—viz. that of Morgan *v.* Boys. The testator in this instance died, leaving by his will a large fortune to his housekeeper. The will was disputed by his relatives, on the ground that it bore intrinsic evidence of his not having been in a sane state of mind. After having bequeathed his property, the deceased directed that his executors should cause some part of his bowels to be converted into fiddle-strings, that others should be sublimed into smelling-salts, and that the remainder of his body should be vitrified into lenses for optical purposes! He further added in a letter,—" the world may think this to be done in a spirit of singularity or whim," but he expressed himself as having a mortal aversion to funeral pomp, and he wished his body to be converted to purposes useful to mankind. Sir H. Jenner, in giving judgment, held that insanity was not proved; the facts merely amounted to eccentricity, and on this ground he pronounced for the validity of the will. It was proved that the deceased had conducted his affairs with great shrewdness and ability; that he not only did not labour under imbecility of mind, but that he was treated as a person of indisputable capacity by those with whom he had to deal. The best rule to guide the court, the judge remarked, was the conduct of parties towards the deceased; and the acts of his relatives evinced no distrust of his sanity or capacity.

In this instance, the testamentary disposition was contrary to the usual order of succession, as it is in all cases of disputed wills; but there was no delusion accompanying it. The will was drawn up by the deceased himself, and bore evidence of a reasonable mind, notwithstanding the introduction of his absurd notions. It is proper to remark that the deceased had always been noted during life for his eccentric habits, and had actually consulted a physician upon the possibility of his body being devoted to chemical experiments after death.

The most difficult medico-legal questions connected with this subject are those which relate to responsibility for crimes on the part of persons labouring under *homicidal monomania.*

"It is not easy to convince a common observer that a man who enjoys the sound exercise of reason upon all subjects except one should not be considered accountable for any act he may perform, and be liable to punishment if it should be criminal; and it is still more difficult to convince one unacquainted with the subject, that a man who presents no appearance of intellectual aberration should be compelled to the commission of deeds which are not only criminal, but revolting to humanity, and which he himself laments when the anguish has been quieted by the fulfilment of the desire. The subject is one of very great difficulty, and it is not to be concealed that the existence of such a disease as 'instinctive monomania' has been altogether denied. It has been called by a celebrated counsel a modern resource, which would be extremely convenient to snatch a criminal from the just severity of the laws, or to deprive a citizen of his liberty, and would soon convert our lunatic asylums into prisons." (p. 209.)

That those who have not considered the subject should find a difficulty in admitting this form of insanity is not at all surprising, although there must be an equal difficulty in allowing that the crime of murder should ever be committed without a motive by a reasonable being. The absence of all motive, and of any attempt to conceal the crime after its perpetration, are the chief characters of this kind of monomania according to Dr. Pagan. Homicide under the influence of ungovernable passion is not excused by law, yet here there cannot be said to be a motive, at least such a motive as usually incites a person to destroy another (ira furor brevis). The author thinks that in the case of passion there may also be no attempt to conceal the crime, but the passions are, at least in most cases, capable of being controlled. We put little stress on the first point (concealment), but the second is of more importance, yet the law here makes such allowance for the difficulty of controlling passion, as to reduce the crime from murder to manslaughter. In some cases there is a mixed condition of passion and insanity, arising from long indulgence or peculiarity of disposition, in which the fit of passion appears to be a state of temporary insanity, differing only from mania in its short duration, and being speedily followed by the return of reason and the exercise of the intellectual faculties. Some have called this *iracundia morbosa.* In persons labouring under this infirmity anger is rapidly changed, without the slightest reason, into absolute and uncontrollable fury. Evidence of this state is often given on trials for murder; but it would be difficult to produce a case in which the proof of it had led to the entire irresponsibility of the accused. The party is generally convicted of manslaughter.

The existence of homicidal monomania can only be decided by an appeal to facts. Allowing that it does exist, we must admit that he who labours under it should be held, to a certain extent, irresponsible for his acts. In all criminal cases, the test by which the jury have to try the state of mind of the prisoner is the following,—whether, at the time the act was committed, the prisoner was incapable of judging between right and wrong, and did not then know that he was committing an offence against the law of God and of nature. This is the rule commonly laid down by our judges as the spirit of English law; but we agree with the author in thinking that any test more fallacious in its general application could not be possibly conceived. Further, the law always presumes the competency of parties, and the question is presented to a jury upon the negative, which must be established on the behalf of the prisoner. In this respect there is a wide difference between the civil and criminal law;—if a person has once been found lunatic by a commission, all his civil acts are considered to be those of an insane person until his lunacy is legally revoked, or the clearest proof of a lucid interval is adduced: but if he have been guilty of any criminal act, the circumstance of his having been already found lunatic by a commission, or of his having been for years notoriously mad, will avail nothing: he is put upon his trial, and the question of his insanity in the legal sense above mentioned, at or about the time of the act, must be again gone into. The criminal law presumes that every person arrived at the age of discretion is sane, and is accountable for his acts, unless the contrary appear. The legal proof of insanity does not here rest on the evidence of the existence of delusion, or even on the connexion of that delusion with the act itself, but on the incapacity of the party at the time to distinguish whether the act he was committing was criminal and contrary to the law of God and man. We do not complain of the law for thus jealously enquiring into the circumstances connected with the violent death of a human being, but we do complain of the setting up a false test for criminal responsibility, since the effect of this must be not only to add to the number of victims, by executing persons morally irresponsible, but to make punishment purely vindictive. It is notorious that insane patients often commit acts which they know to be wrong, and of the criminality of which they are perfectly aware. They have been known to murder others in order to receive the punishment of death from the hands of the law, and therefore they must have known that the act which they were perpetrating was an offence against the law of God and man. In short, the criminal character of the act has often been the sole motive for its commission. This fact, which is totally independent of the admission of the existence of such a state as homicidal monomania, appears to us to establish clearly that our criminal law is acting upon a dangerous error, that the test required for establishing criminal responsibility is founded upon false notions of the state of the faculties in insanity, and that thus many really insane persons must, by its enforcement, be put to death, without the law having the justification of preventing, by the terror of example, the perpetration of similar acts. But the error has a wider influence than this: it is, we believe, the main cause of such a state as instinctive homicidal madness being rejected as a plea in law. In this state the individual, as we know, is generally aware of what he is about; he knows that in killing another he is committing a crime, and if this is not admitted at once by his own confession, it is easily to be inferred from his general conduct. All we learn from him is, that he was ruled by an impulse which his will could not control, yet the law does not in any case admit this as an exculpatory plea, and it thereby denies the existence of such a state as diseased volition or diseased comparison. It appears to us that the true test for irresponsibility should be, not whether the individual knew that what he was doing was criminal, but whether he had sufficient power of control to govern his actions. It might be fairly asked, how is such a test to be applied, so as to ensure protection to society, and prevent injustice to those labouring under mental disease? We can only reply, that we must judge from circumstances, a practice now daily followed in the determination of the shades of guilt by which murder passes into manslaughter. The sole difference between these two crimes rests, in a large number of instances, upon the power of self-control in the accused party. This is always inferred from circumstances, such as the mode of attack, the presence or absence of reasonable motive, and the time which has elapsed during a quarrel before the fatal blow is struck. It cannot then be urged that we are here suggesting a novelty for a test of legal responsibility.

We must, however, take the liberty of observing that, whatever substitute be adopted, some alteration of the law is absolutely required. Many eminent law-writers advocate the rule now followed, because it prevents juries from being embarrassed by any technicalities respecting the import of the term insanity. It certainly has this advantage, and the only objection to it is that it does not go far enough: while it leaves many of the insane irresponsible, it does not leave all; it therefore operates unjustly. If insanity consisted, as is often vulgarly supposed, in a total absence of reason, and an unconsciousness of actions, this legal rule would be sufficiently correct; but a very slight practical acquaintance with the subject will show, that this view of the malady is only applicable to a few cases, and never ought to enter into any system of criminal jurisprudence.*

The mere proof of *delusion* in a criminal does not exculpate from crime. In 1834, a man was tried for shooting at another with intent to murder. The medical witness stated, in answer to evidence, that the prisoner had previously supposed he had seen an apparition, but this, in his view, was only a proof of great weakness of mind or intellect, and not of derangement. Here we see a distinction drawn by one who had evidently never thought of the subject prior to the examination. We offer no opinion on the responsibility of the party in this case, but it would have been curious to have seen, by a rigorous cross-examination, how the medical witness would have justified his statement.

It would be going too far to say that all persons who have experienced delusions should be held irresponsible for their acts. Where the ordinary motives for crime exist, with premeditation, precaution, and concealment, then we consider the interests of society would be best protected by the infliction of punishment. But is it absolutely necessary for the establishment of an exculpatory plea that the delusion should be connected with the act? Where there is this connexion, as where a man kills another under the false impression that he has sustained the severest injuries from him, then we think there can be no doubt of irresponsibility: this is one of the least difficult forms of homicidal madness to the medical jurist. The question, however, is not always so simple as this: a man who labours under many delusions may commit a crime, and yet by no process of reasoning can we establish the least connexion *in our minds* between the act and any one of these delusions. How is such a case to be disposed of? In defending Hatfield for shooting at the king, Lord Erskine argued, that to exempt from responsibility there must be a close connexion "between the delusion and the act,—the prisoner's act must be the immediate unqualified offspring of the disease." This doctrine is so inapplicable, that were it rigorously acted on few lunatics tried for murder would escape the punishment of death. Indeed, it is surprising

* Several cases have occurred of late years, which show that this legal doctrine cannot be always practically enforced. Jonathan Martin, the incendiary, was perfectly conscious that he was doing wrong when he set fire to York cathedral. He admitted that he was doing wrong, according to the law of man, but he had the command of God to do the act. This man was rational upon all other subjects. He was acquitted; and hence we must draw one of two inferences, either that he ought to have been hanged as a criminal, or the test of responsibility uniformly assumed by the law is wholly inadequate to the purposes of justice. Vide also the case of Rebecca Hodges, tried at Warwick in 1809.

that, in making the assertion, it did not occur to the mind of this excellent lawyer, that the act of an insane person might be strictly connected with his delusion, without our being able to discover the connexion, and that it must be the height of inconsistency to test the motives which govern the conduct of a lunatic by those which would guide a reasonable being.

This doctrine of Lord Erskine's has not, so far as we know, ever been acted upon. It would be easy to adduce cases, where persons have been acquitted on the ground of insanity in a charge of murder, notwithstanding the want of any apparent connexion between the delusion under which they laboured and the crime, and this even where the motive seemed to be revenge. On the other hand, it has been held by judges that, although the mental delusion might be connected with the crime, and actually stimulated the party to commit a murder in revenge for an imaginary injury, yet if he knew he had no right so to revenge himself, he would be criminally responsible. We hereby learn that in all these cases the existence of delusion and its connexion with a crime have less to do with the responsibility of a really insane person than his capacity to distinguish right from wrong at the time of its commission. This is a practical enforcement of the doctrine advocated by the attorney-general in Hatfield's case, in opposition to Lord Erskine,—a doctrine sanctioned by the authority of Lord Coke and Lord Hale, that "to exempt a man from crime, there must be a total deprivation of memory and understanding."

Dr. Pagan, with most medico-legal writers, argues that homicidal madness should not be admitted as a plea of exculpation, "except upon the clearest proof derived, in the majority of instances, from the absence of precaution in the mode of committing it, and of the usual criminal motives, conjoined with the positive proof of the existence of the disease."* (p. 214.) In another place he says: "In no case, in which a criminal motive can be discovered, perhaps, would the medical jurist be justified in giving an opinion that the person was irresponsible." (p. 210.) Then again, at page 233, we find him asserting, in commenting on a particular case, that "the presumed motive was altogether inadequate to account for murder." We have some doubt how far the mere absence of precaution in the commission of crime should be allowed to guide our judgment, since there are many instances in which sane criminals show great want of foresight and precaution, and others again in which those who are really insane display the greatest cunning; thus they will sometimes carry a weapon for weeks before they use it against their intended victim, and calculate the time and opportunity for destroying him with the greatest precision.†

The absence of the usual criminal motives is a matter of much greater importance; indeed, this is the only evidence of the existence of one class of cases of homicidal mania, namely, those in which "no trace of partial insanity can be detected, in which there is no hallucination, no delusion, no fixed idea, but in which the person is impelled by an irresistible impulse to shed blood, which he is altogether incapable of resisting by the exercise of a depraved or weakened volition, in which no motive for crime exists, in which no act, ulterior to the murder, is

* Hatfield's case, contrà. † Vide Hatfield's case.

performed, not even, perhaps, an attempt made to conceal it, or the individual's participation in it, and in which none of the usual incentives to crime can be detected." (p. 214.) Some writers have called this moral insanity, or instinctive homicidal mania (mania sine delirio), and have accumulated instances of fond parents murdering their children, husbands their wives, and servants their masters. The difficulty to the admission of such a state appears to us to consist in the fact that the insanity is pleaded for the crime only, that it did not exist before or after its perpetration, and that it may be thus converted to a specious means for totally exempting criminals from punishment. Sometimes murder is committed on a sudden and uncontrollable impulse, like suicide; but perhaps more generally, if we are to trust to the confessions of criminals, the idea of destruction has long dwelt in the mind, has been long resisted from a conviction of its unlawfulness, but finally, in a moment, it has triumphed. Dr. Conolly has observed,* that the degree to which this feeling admits of resistance is often a very important question. The law harshly judges of this power of control by the conduct of the individual before and after the perpetration of the act; but psychologists are well aware that no evidence may be derived from such an examination, to support the ordinary idea of the existence of insanity. The individual may have experienced the feeling, but have sufficiently mastered it to prevent it having the least influence on his general conduct.

The existence of such a state as instinctive homicidal madness is involved in the question, Whether a sane person is likely to destroy one to whom he is fondly attached, without the least criminal motive? We think not; and the perusal of the cases accumulated by Esquirol, Prichard, and others, and quoted by Dr. Pagan, appears to us strongly to bear out this view. The law asks,—Do they not know at the time that what they are doing is wrong? We reply, in many cases they do; but would suggest that the true question should be,—whether they have the power to resist the horrid impulse which leads to the perpetration of the crime? if they have, let them be convicted; if not, let them not be condemned as we should condemn a reasonable being. We must remark, however, that it is doubtful whether, in any case, there is an entire absence of motive; all we can say is, that the usual motives of revenge, robbery, or for the concealment of other crimes, are not apparent. The homicidal monomaniac is often induced to destroy his children to save them from want, or to convert them to angels, or his motive springs from some other extraordinary delusion. But it may happen that the person murdered was a stranger to the prisoner, and had given no provocation whatever; here, as in the former case, the only reason to suspect insanity might be the act itself. How would such a case be met by the law? The following instance will show: Soon after Bellingham's trial, a man was tried at Warwick, who rushed into a room where there were persons whom he had never seen before. He killed one with a knife which he carried, and gave another seven wounds. He then rushed out and ran into a river, brandishing the weapon with which he had inflicted the injury. Under the judge's directions the jury

* Indications of Insanity, p. 339.

negatived the plea of insanity.* This seems to have been a case of homicidal madness, for there was no motive whatever for the act, and it is most probable the crime was committed under a delusion. There is a more difficult class of cases, where the crime is committed for a motive, which to a rational mind seems inadequate,—as where one man murders another for the sake of obtaining from his person an insignificant sum of money, or an article of little or no value. These cases are of continual occurrence: we do not see how the medical jurist can interfere with them, since it is impossible to define exactly the degree of adequacy in motives. If the criminals have used no reasonable effort to control these petty incentives to the commission of heinous crimes, they appear to us to become justly the subjects of temporal punishment.

The cases quoted by the author in illustration are chiefly derived from foreign sources; and we are glad to find by these, that the proof of premeditation and precaution, with a perfect consciousness of the criminality of the act committed, have not, in some parts of the continent, been deemed incompatible with the admission of insanity as a plea. But we should have liked to see more illustrations drawn from the practice of the English law, since they are extremely numerous. Scarcely a circuit passes without many such occurring; for the plea is perhaps one of the most frequent in trials for heinous crimes. Among many that have occurred within the last few years, we might point to that of Greensmith, who was tried on the Midland Circuit, in July, 1837, for the murder of four of his young children, as one well calculated to fix the attention of all English medical jurists. This man, who was of industrious habits, and an affectionate father, fell into distressed circumstances, and destroyed his children by strangling them, in order, as he said, that they might not be turned into the street. The idea only came to him on the night of his perpetrating the crime. He left the house and went to a neighbour's, but said nothing of the murder until he was apprehended the next day, and taken before the coroner, when he made a full confession. After he had destroyed two of them in bed, it appears he went down stairs, and remained some time. Thinking that he might as well suffer for all as for two, he returned to the bed-room, and destroyed the two whom he had left alive. He shook hands with them before he strangled them. Not one of the witnesses had ever observed the slightest indication of insanity about the prisoner. He made no defence, but several humane medical practitioners voluntarily came forward to depose that he was insane.

The surgeon of the gaol had observed that there were many appearances of derangement about him. He was feverish, complained of headach, and had been subject to disturbed sleep, and sudden starts, ever since the death of his wife. He spoke of the dreadful crime he had committed without the slightest excitement; and the witness was about to state the general conclusion at which he had arrived, when the judge (Mr. Justice Park) interfered, and said, that the witness must not state his opinion; it would be for the jury to draw their inference from the facts stated by him. In answer to a question, the witness said that he had heard enough of the evidence to satisfy him that the prisoner could not have committed such a crime as this, and be in a right state of mind.

* Prof. Amos.

The judge observed that was no ground to warrant such a conclusion. The question was, did the prisoner know right from wrong at the time, as every person might be said to be insane in some sense of the word, who committed a great crime. Dr. Blake, physician to the Nottingham lunatic asylum said he was satisfied that the prisoner laboured under a delusion of mind. His sister and grandmother had been under his care, the former for entertaining a similar delusion, namely, that of destroying herself and children. The judge declined receiving this evidence. He then summed up the law, as laid down in books with respect to what would constitute such a case of insanity, as would render a person irresponsible for his acts. The jury found the prisoner guilty, and sentence of death was passed on him. The prisoner displayed no emotion whatever during the proceedings. This unfortunate person would undoubtedly have been executed, had it not been for the active interference of Dr. Blake and others, who drew up a full representation of the facts, forwarded them to the secretary of state, and procured a respite on the ground of insanity.*

We have here selected one English case of recent occurrence, to illustrate the state of English jurisprudence in regard to the responsibility of the insane for crimes. In it too we have a complete history of homicidal monomania. We doubt whether any person who has attended to the subject of insanity could read the above case without feeling convinced that the prisoner had committed the act while labouring under a delusion; and that, upon the bare proof of the facts, he ought to have been held irresponsible as a murderer. Yet we see this was denied, because the prisoner was evidently conscious he was committing a criminal act. He killed his children in order, as he expressed it, that he might himself "suffer for it." We are surprised it did not occur to the learned judge who tried this case, that if the facts did not constitute a case of insanity according to the legal definitions, those definitions must involve some error, and were not adapted to the purposes of justice.

There are several points of importance in the above proceedings relative to the medical jurisprudence of insanity; and, as they may affect the duties of medical witnesses in future cases, we shall here briefly advert to them. The judge, it will be observed, would not allow the medical witness to state his opinion whether the prisoner was insane or

* As we should be sorry to misrepresent the views of the learned judge on a question of such importance, we subjoin a part of his charge to the jury, from a letter published on the subject by Dr. Blake:

"The physician (Dr. B.) who had stepped forward to urge the plea of insanity on behalf of the prisoner, had stated a doctrine very opposite to this," (alluding to Sir M. Hale's definition of criminal responsibility,) "and one which was altogether inadmissible in a court of justice; and which gave him some surprise, when he heard it advanced by a gentleman occupying so important a station in society. Nothing could be more contrary to law than to infer insanity from the very malignity and atrocity of the crime. It was true, that such crimes could never be committed by men who were in possession and control of a right reason and a proper mind: but it was his duty to inform the jury that the complete possession of reason was not essential to constitute the legal any more than the moral responsibility of man; it being merely necessary that the party should have sufficient knowledge and reason to discriminate between *right* and *wrong*."

A judicious notice of Dr. Blake's letter appeared some time since in a contemporary journal. (*Med. Chir. Rev.* vol. xxviii, p. 84.)

not. It has been ruled in the criminal law that a witness is at liberty to say whether such and such symptoms, proved by other witnesses, are in his judgment those of insanity or not; but some of the judges have held that he cannot be asked whether the prisoner is insane or not, and still less whether the act for which he is tried be an act of insanity. This is a point which it is considered must be decided by the jury alone. We cannot see the policy of this decision; why, for example, an individual above all others the best fitted to form an opinion in such a case should be restrained from expressing it. The jury may or may not coincide in the view taken by the witness; a circumstance which would depend upon the reasons assigned by him for his opinion, but why the prisoner should not have the benefit of this opinion it is difficult to understand. In an ordinary case of murder by wounding, it might as well be ruled that the witness is not to be asked whether the deceased died of the particular wound, but the jury must infer this from his evidence. It is also not a little curious that, in a civil case involving the question of the insanity of a party,—as before a commission, and on suits relative to disputed wills and marriage contracts, the medical witnesses are often expressly called upon to give their opinions on the state of mind of the party, and whether his acts were those of insanity. Is life of less value than property, that a medical opinion should be actually required in the one case and entirely rejected in the other?

A second point worthy of notice in this trial is that the judge refused to receive the act of the prisoner as furnishing any evidence of his insanity. Now as, in many instances of homicidal mania, there is nothing but the act itself indicative of insanity, so must we consider this as substantially rejecting a large number of cases from that protection which a humane system of jurisprudence is bound to afford. But why is a civil act taken to furnish evidence of insanity, while a criminal act is not? In judging of the validity of a will or contract, it has been repeatedly held by our best ecclesiastical judges that the act itself furnishes the best and sometimes the only evidence of the insanity of a party. There can, we think, be no doubt of the truth of this position, and we do not see why the rule should not be equally applicable in criminal cases.

The judge objected, it will be seen, to receive the evidence of Dr. Blake, as to the existence of insanity in other members of the prisoner's family. Here again we meet with what is represented as being an established rule of law, applied both to civil and criminal cases; namely, that proof of other members of the same family having decidedly been insane is inadmissible as evidence. If we can trust to the observations of the most experienced men in all countries, there is no doubt that, beyond all other diseases, insanity is hereditary. According to some, upwards of one half of the cases which occur are to be traced to an hereditary origin. With the admission of this well-ascertained medical fact, it seems inhuman to reject such collateral evidence, in obscure cases of suspected homicidal monomania; since, if any error be committed by its admission, it will certainly be on the side of mercy.*

Those who, like Dr. Pagan, require that cases of this kind should be

* In this instance there was not merely an hereditary tendency; but, as Dr. Blake has stated, there had been a perfect similarity of delusion in the prisoner's sister.

dealt with in a more humane and liberal spirit than we at present find manifested by our law, are well aware of the danger which might follow by too readily admitting a plea so easily set up and so difficult to disprove if once admitted. It is not of course imagined that individuals once proved to have committed such heinous crimes should be set free, for this would endanger the safety of society; all that we might expect is that their lives should be spared, and that they should be confined in asylums. This might by some be considered as a punishment which would not deter others from acting in the same way; but an objection of this sort applies to crimes committed by lunatics in general, and, although we could not thereby prevent others from committing murder, we should prevent the same individual from doing further mischief.

But murder is not the only case in which "a perfect consciousness of the quality of an act, and the power of resisting the impulse to commit it, independently of the usual passions, are not invariably combined." Incendiarism and theft have been considered by many medico-legal writers as now and then resulting from a monomaniacal propensity. The circumstances requiring investigation, in these instances, are the same:

"We must ascertain whether the person has previously been insane,—whether he had taken precautions to ensure the perpetration of the crime, and avoid detection,—whether these precautions were adopted before or after the act; and, above all, whether it seems to have been the consequence of the usual inducements to crime: these, with the positive evidence of the existence of delusion, or of some unusual and unnatural impulse, will lead us, in most instances, to a sound conclusion as to the responsibility of the individual for the act in question." (*Pagan*, p. 252.)

Cases of *dementia* form a frequent ground for disputing the validity of deeds; and here we meet with an additional difficulty; for persons in this state are easily subject to control, and are liable to be influenced by interested persons placed about them. The imbecility of age is often adduced as a ground for rendering a will invalid; but here the facts required to be proved are, that the mental weakness was such as either to render it impossible for the individual to know the nature of the act in which he was engaged, or to subject him to the improper control and interference of others. In these enquiries, it must be remembered that the powers of the mind may sometimes be suddenly restored, and the individual acquire a disposing capacity just before death. An attack of acute disease has been known to produce this effect in dementia. (*Pagan*, p. 266.)

Wills made *in extremis* are always properly regarded with suspicion, and many instances of their revocation might be adduced. It is generally impossible to obtain satisfactory medical evidence of the state of mind of the testator. In examining into the capacity of a person under these circumstances, we should avoid putting leading questions, namely, those which suggest the answer, yes or no. Thus, a dying man may hear a document read over, and affirm that it is agreeable to his wishes, without understanding its purport. It is not satisfactory, unless he himself dictates the provisions of the document, and is able to repeat them: if he does this, accurately, there is no doubt of his having a disposing mind. No general rules can be applied to these cases,—some persons retain the perfect enjoyment of their faculties to the last, even through a lingering disease; in others, where the disease affects the brain or nervous system, as in fevers or acute diseases, which speedily cut short life, the intellectual powers are commonly obscured. To prevent fraud, one rule of law is uniformly acted on in all these cases, namely, where incapacity is alleged on the part of a testator, the evidence to establish it must be clear and distinct, or the objections to the document will be disallowed. We must regret that Dr. Pagan, although he admits this question is often one of the greatest difficulty to the medical jurist, has not produced some cases to illustrate the subject. Many such have come of late years before our courts, and there is scarcely one which does not furnish instruction and interesting matter for reflection. *Brevis via per exempla, longa per præcepta.*

Persons labouring under confirmed dementia are irresponsible for their acts. The only crime of which they are commonly guilty is theft. They seem to have an imperfect sense of right and wrong, and do not sufficiently distinguish between what belongs to themselves and others. They are notorious for hoarding up large quantities of useless articles, and laying their hands upon all kinds of objects, without regard to their quality or value. They seldom commit murder or any grave outrage, except in those instances where dementia alternates with epilepsy, and is succeeded by paroxysms of violent mania.

The remarks on *idiocy* and *imbecility* are sound and judicious. Imbecility is nothing more than a minor degree of idiocy; it is a condition in which there is "a limited manifestation of the intellectual and moral faculties, the consequence of imperfect organization, or in which the development of the mental powers has been prevented in consequence of some disease of the brain in the early periods of life, before education and experience had produced their proper results." (p. 288.)

Idiocy is a condition in which the medical jurist has little concern. The mind of the idiot never having become developed may be regarded as destitute of ideas; hence there can be no doubt of their utter incapacity for undertaking the social duties of life. Cases of imbecility, however, are among the most embarrassing which occur, since the individual is not so devoid of intellect as to be incapable of receiving instruction; and he may have attained enough knowledge to have discharged some of the duties of life, although in an imperfect manner. Cases of this kind have rarely come before a court of law without giving rise to the most conflicting statements among the medical witnesses; and we have no hesitation in saying that they have often reflected disgrace on the profession. This difference of opinion has not always depended upon different degrees of experience in the witnesses, for where opportunities of observation have been equal, it is by no means unusual to find diametrically opposite views expressed of the state of mind of a party. Dr. Pagan cites the case of David Yoolow as an instance of this kind. Our readers will find a full report of this interesting case in the Eleventh Number of this Journal (July, 1838). The attempt there made was to prove idiocy: the medical evidence clearly established imbecility in our view; but the proof of this state was not deemed sufficient in law. No notice is taken of the celebrated case of Miss Bagster, which, as we shall hereafter see, affords an instructive example of the sort of medical evidence which is delivered on lunacy commissions.

We cannot be surprised at the conflicting verdicts which are returned on these enquiries when we meet with such conflicting medical evidence. One physician tests one faculty, another, another; each has his own definition of insanity, and each forms a different opinion of the state of mind of the subject of enquiry. What we must strongly condemn on these occasions is the narrow and restricted manner in which the examination of the alleged imbecile is conducted. The witnesses for the commission do not go to test the state of mind of the party, so much as to discover what they may deem proofs of insanity: those against the commission adopt the opposite line of conduct; and thus the medical witnesses become as much partisans in the suit as the counsel engaged for and against the lunatic. We do not wish to express a harsh judgment on these cases; but we confidently appeal to all who have closely attended to the medical evidence on commissions, whether a strong bias is not in general discoverable in it. Witnesses may differ in opinion from entertaining opposite views of the same question, each having his own standard of comparison: for, as it has been well remarked, our judgments are like our watches—none go just alike; yet each believes his own: but we are satisfied that the great cause of the evil is the appointment of medical witnesses by the parties themselves. We fully agree with the author in thinking that "a man, even unknown to himself, with the purest intentions and most perfect rectitude, insensibly leans to the side upon which he has been employed. He is disposed to find that party in the right, and draws conclusions too hastily from premises too narrow." (p. 301.) There is often another cause for this difference in operation, which a medical jurist will do well to remember. Persons labouring under imbecility are soon irritated; they are easily persuaded that they are ill used and persecuted; and when they are questioned by parties, who are represented as their enemies, they lose their self-command, and are no longer able to answer questions which, under their ordinary state of composure of mind, they would reply to with perfect accuracy. (p. 302.)

We entirely dissent from the author's view, at least so far as English practice is concerned, that there is "a repugnance on the part of juries to interdict persons of decidedly weak intellect;" and we would refer among others to the case of Miss Bagster, as a direct proof of the contrary. This lady showed by her examination, and we learn the same facts from the medical evidence brought against her, that although weak in mind, she possessed a far greater degree of mental power than existed in the case of Yoolow. Yet she was found to be of unsound mind, while Yoolow was pronounced sane. We are obliged to declare, from the examination of such cases, that the manner in which the disposition of property will be affected by the verdict is as much looked to by the jury as the actual state of mind of the party.

It has been properly inferred that the different opinions expressed by medical men on these occasions cannot arise out of their evidence as *medical* practitioners; but that these are cases in which non-medical men and all persons of common sense must be equally capable of judging. The censure conveyed in this remark is but too well merited, and witnesses should reflect whether in the end this conduct may not lead to the total abandonment of medical evidence on commissions. Under the present system, practitioners are certainly placed in an embarrassing position. The party who summons them to examine the supposed imbecile fully expects that the examiner will give an opinion favorable to his view; indeed, it is often looked upon as bought and paid for. Unless the medical witness firmly exerts his independence in this matter, he may be the means of inflicting irreparable injury. But there are many worldly inducements which may make him hesitate, and he who hesitates is lost. The obvious remedy is that the witnesses should be men of character, selected for their knowledge of the subject, and made as perfectly independent of either party as the commissioners themselves.

We cannot close these remarks without enforcing on the medical jurist the necessity for his conducting the examination of an alleged imbecile with a proper regard to all the circumstances of *age*, *society*, *education*, and *general conduct*. We must first learn what the party has been taught, what instruction he has received, and how far he has profited by his education. We have already observed that the questions put to test mental incapacity are sometimes of the most absurd character; and the inference deduced from the answers equally so. No one can be set down as an imbecile because he is ignorant of that which he has never had the opportunity or disposition to acquire. We have always been at a loss to understand on what principle the arithmetical test has been introduced on these occasions. Some are not capable of being taught arithmetic, others acquire it in different degrees, while some have a mental arithmetic, without being able to express it in figures. If the capacity to manage affairs rested solely upon a knowledge of arithmetic, we are satisfied that many now go free who ought to be put in custody. A girl of sixteen is asked to calculate the rate of interest in the funds upon a given sum, and because she cannot answer the question this is a proof of her imbecility and consequent incapacity to manage her affairs! We know that there have occasionally been put to alleged imbeciles questions in numbers which other medical witnesses for the same commission have not themselves been able to answer. In our opinion, the questions should always be confined to what there has been the opportunity or inclination to learn; or otherwise we might be continually confounding mere ignorance with imbecility. Perhaps the best test of mental capacity in these cases will be found in determining the degree to which the individual has shown himself capable of receiving instruction.

The criminal responsibility of imbeciles involves serious considerations, since, here more than in any other case, an erroneous verdict may be returned, and capital punishment unjustly inflicted. The shades of imbecility are so various that it is difficult to draw a line of demarcation between it and perfect sanity. The practice of our law leads, moreover, to the inference that an imbecile who has not sufficient capacity to manage his affairs or property, may still come within the legal pale of criminal responsibility, i. e. he may have a moral perception of right and wrong. This test we must condemn, as even less applicable in these cases than in homicidal madness; for we never can presume "that the moral sense is perfect when the intellect is weak, and has never been developed." (p. 305.) We have no means of ascertaining, even with the acknowledgment of the party, that he fully comprehends the meaning of these terms. The fear of punishment probably operates as the chief preventive to crime in these cases, and not the moral perception of the iniquity of the act. The same may be said of the sane criminal; but the difference lies in the presence or absence of a power of self-control. This, which we presume to be present in the sane criminal, is absent in the imbecile. Our opinion, says the author, of the responsibility of a person of acknowledged imbecility, " will be formed from personal intercourse—from the history of his life—from the circumstances connected with the commission of the crime, rather than from abstract notions of what his perception of right and wrong may amount to, which it is nearly impossible, I think, to know." (p. 306.)

The degree of education received, the kind of instruction, and the progress made by him, are points which require attention. On the other hand, his knowledge of the criminal character of the act may be inferred from the manner in which the crime has been perpetrated; from the fact of precautions having been taken to ensure secrecy in its perpetration, and safety afterwards; from its having been committed to facilitate the perpetration of another crime; from his having invented an artful story to free himself from responsibility, and throw it upon another; all these are circumstances from which we may infer a moral perception; and in such cases the accused ought undoubtedly to be held responsible as a sane criminal.

Imbeciles, it is true, sometimes display great premeditation and precaution as well as criminal motives in their actions; here a conviction generally follows; but it is questionable whether it would not be sounder policy to substitute confinement, as Dr. Pagan observes, for a punishment which can make no moral impression.

From the manner in which the subject of insanity is usually studied, the medical witness must be careful of not falling into an error which has been more than once committed, namely, that of presuming that a criminal must be of sound mind, merely because his case cannot be arranged under any of the four great divisions of insanity. "These divisions are adopted for the classification of facts, and as a means of facilitating their study; but they cannot set limits to nature; and it is frequently a matter of no little difficulty to determine under what division of insanity a case should be classed." (p. 321.) In some instances we meet with characters which appertain to all the divisions.

The medico-legal relations of the *deaf and dumb* do not require much consideration. An uneducated deaf and dumb person is altogether irresponsible for any action, civil or criminal. A deaf and dumb girl was tried for the murder of her child at Glasgow. The trial was stopped on the objection of her counsel that the prisoner could not plead, for she did not know the meaning of guilty or not guilty. There was no doubt of her moral responsibility, for premeditation, precaution, and a criminal motive were apparent from the facts detailed. An educated deaf and dumb person, one who can be made to understand by signs, would undoubtedly be subject to trial, and might be held responsible according to the circumstances proved.

Under a similar limitation, a deaf and dumb person may act as a witness, or may contract matrimony, since his consent is valid and may be given by signs, provided there is an evident understanding of their meaning.

The last condition noticed by the author is that of *somnambulism* or sleep-walking, in which there is a power of exerting the muscles of locomotion, and performing many acts while the individual is completely deprived of his senses. Supposing that a person while in this state commits a crime, is he to be made responsible? Dr. Pagan, with other medical jurists, thinks not; but professes himself unable to suggest any means by which the reality of this state, at the moment of perpetration of the act, may be established. Admitting that the criminal action committed during sleep is one which has been premeditated while the individual was awake, we doubt whether we should be ever justified in inferring irresponsibility. In a case of this kind, a medical man might admit the possibility of the act being thus committed unconsciously; but the court would have other facts to guide it in its decision—as the previous relations of the parties, the presence or absence of motive, with many other circumstantial points, which might even render a medical opinion unnecessary.

"Murder has not unfrequently been committed in that half unconscious state at the moment of awakening from sleep—in the intermediate state between sleeping and waking." (*Pagan*, p. 326.)

There are several cases of this kind on record, and it involves a serious question for the medical witness. A man may be awakened by a frightful dream, and, under the temporary persistence of the delusion, kill some one who is near him. It is certain that suicide is often thus committed. An individual will go to bed without the least idea of self-destruction, will suddenly awake and destroy himself. Very recently, an old lady, residing in London, awoke in the middle of the night, went down stairs, and threw herself into a cistern of water, where she was found drowned. Admitting, then, that suicide may be thus committed, we shall have no hesitation in believing that when the means are at hand, some such delusion may lead to the perpetration of murder. But the responsibility of a party will never rest upon the bare possibility of the occurrence of this state. Other facts will be sought for, and must be forthcoming, to show whether there existed a criminal consciousness of the act.

A person, awakened suddenly from a deep sleep by another approaching, may destroy life without being fully aware of what he is doing. Dr. Pagan thinks a person could not be held responsible under these circumstances; but we think no general opinion can be given on such cases.

The following occurred to our knowledge within the last few years: A pedlar, who was in the habit of walking about the country armed with a sword-stick, was awakened one evening, while lying asleep on the high road, by a man suddenly seizing him and shaking him by the shoulders. The man, who was walking by with some companions, had done this out of joke. The pedlar suddenly awoke, drew his sword, and stabbed the man, who soon afterwards died. He was tried for manslaughter. His irresponsibility was strongly urged by his counsel, on the ground that he could not have been conscious of his act in the half-waking state. This was strengthened by the opinion of the medical witness. He was, however, found guilty.

Such a state of course is easily feigned and with difficulty detected.

We here conclude our notice of Dr. Pagan's book: we need hardly say we strongly recommend it to our readers. The views which he takes of the difficult questions which the medical jurisprudence of insanity involves are just, and consistent with the present state of knowledge. The work evinces throughout deep reflection, and a perfect knowledge of the subject on the part of the author.

2. We shall now proceed to offer some remarks on Dr. Ray's treatise, taking care to confine our observations to those points which may throw any new light upon the subjects already discussed.

The American writer commences with some preliminary views on the jurisprudence of insanity, and he adopts a tone of strong but well-merited censure in criticising the conflicting opinions of legal authorities in Great Britain and the United States. He justly refers the present crude notions which exist on the subject to the fact that the legal relations of the insane were laid down long before physicians had obtained any accurate notions respecting the state of insanity. Modern discoveries have been rejected as the innovations of theorists, and have been set aside on the old principle "*Omnis innovatio plus novitate perturbat, quam utilitate prodest.*" We believe him to be correct in stating that

"One reason why the criminal law of insanity has undergone so little improvement in England is probably that the accused, not being allowed counsel to speak in their defence, except in trials for high treason, the officers of government have always been at liberty to put their own construction on the law, and urge it on the jury as the only correct one, without being contradicted or gainsayed. Thus the old maxims have been repeated year after year, and, not being questioned, their correctness has remained undoubted both in and out of the legal profession. Can any one doubt that had those insane criminals who have been condemned within the last half century been defended by an Erskine, many of them would have been acquitted, and a great advance made in the law of insanity, that would have prevented some of those exhibitions of presumptuous ignorance which will one day be universally regarded with feelings of disgust and pity." (*Ray*, p. 25.)

We must here remark that prisoners in this country have been now for some time allowed counsel to conduct their defence in charges of felony; but they have it not always in their power to provide themselves with advocates of equal talent and ability with those employed by the crown to prosecute them.

The author takes much the same view of the legal test of responsibility that we have already taken in noticing Dr. Pagan's work. After asserting that the insane mind is not entirely deprived of the power of moral discernment of the good or evil tendency of actions, he makes the following sound observations:

"If this power of distinguishing right from wrong do really indicate soundness of mind, it may be justly complained that the question of its existence is never agitated in any but criminal cases, while it certainly should be whenever the rights and liberties of the insane are to be invaded. If it is proper to make those who possess this power responsible for their criminal acts, how unjust and absurd is it to deprive them of their liberty, and seclude them from their customary scenes and enjoyments, before they have violated a single human law. Undoubtedly this measure will [would?] be conducive to their good, by taking from them effectually the opportunity of injuring the persons or property of themselves or others; and so it would be for every other unprincipled and reckless individual who bids fair to be a pest to society. But if it is alleged that the latter are morally free, and therefore are personally free, till the commission of some external act, it may be replied that the former on the hypothesis of the law, which makes moral freedom consist in the power of distinguishing right from wrong, have the same claim to immunity from personal restraint. This preposterous distinction between civil and criminal cases gives rise in practice to one of the most curious and startling inconsistencies that human legislation ever presented. While the mental impairment is yet slight, comparatively, and the patient is quiet and peaceable, the law considers him incapable of managing himself or his worldly affairs, and provides him with a guardian and a place in the wards of an hospital; but when the disorder has proceeded to such a height as to deprive the maniac of all moral restraint, and precipitate him on some deed of violence, he is to be considered most capable of perceiving moral distinctions, and consequently most responsible for his actions." (*Ray*, p. 31.)

It certainly does seem eminently absurd that one standard of insanity should be set up for civil and another for criminal cases; or that a man whose civil acts would be ipso facto invalid, should still be made answerable for a criminal act. We have already, however, expressed our opinion of the inconsistency of the law, the "*dictamen rationis*," as it is sometimes called.

We pass over the remarks relative to the fallacy of trusting entirely to the discovery of design and delusion in the conduct of an individual as tests for his responsibility; but we cannot avoid quoting a passage in which a popular error is ably analyzed and refuted. It was an axiom of Lord Hale's that all crime is the offspring of partial insanity:

"And the inference he meant should be drawn from it is that partial insanity furnishes no excuse for crime. It is a curious fact that many benevolent people, in their desire to palliate the sins of criminals, have inculcated the same principle for the purpose of drawing from it a very different inference. . . . . . Says the former, crime must be punished; but all crime proceeds from madness, therefore madness furnishes no exemption from punishment. Says the latter, madmen are not responsible for their criminal acts; but madness is the source of all crime, therefore madmen and criminals are equally irresponsible and exempt from punishment. Which of these two precious specimens of human subtlety can claim the triumph of absurdity it is not easy to imagine. Crime is not necessarily the result of madness, not even when perpetrated under the excitement of fierce and violent passions; in the true sense of the word it is never so, but is always actuated by motives; insufficient it may be, but still rational and definite motives. The misfortune which the criminal is going to avert, the interest which he is going to subserve, the revenge he is about to gratify, the insult or injury he is about to repay, are *real* injuries, and insults, and interests, however much they may be exaggerated, or however disproportionately small to the crime they provoke; and the ends to be obtained by the criminal act are real and have an appreciable value." (*Ray*, p. 48.)

He then proceeds to show that the causes which urge the insane to deeds of violence are generally illusory—the hallucination of a diseased brain, or they may act from no motive at all, but solely in obedience to a blind impulse, with no end to obtain or wish to gratify. Strong passions prevent calm deliberation, but do not destroy the power of self-control in a sane person; hence, in cases of this kind, the law reduces the offence to manslaughter, but still punishes the act as criminal. It is with regret we find, whenever the plea of insanity is urged in a criminal case, that our judges so frequently meet it by the observation that all crime is the result of insanity, more or less: the only clear inference from which observation is that all criminals, sane or insane, should either be let free or all should be punished. It is a virtual denial of the existence of any perceptible difference in the motives or impulses which lead to the commission of crime. Dr. Ray considers that the law in the United States is as loose and vacillating as it is in this country; and ascribes it to the one being exactly modelled after the other. He proposes, as a remedy, that in criminal jurisprudence a rule should be laid down in which, to exempt from punishment, the criminal act should be proved to be connected with the mental unsoundness. (p. 50.) This would certainly partially assist in removing the legal quibbles which invest the subject; but we doubt whether the operation of such a rule would not exclude many who are insane from its benevolent effects. In a former part of this paper, reasons have been assigned to show that this test would operate unjustly: and we do not see why, if there be any alteration made in the criminal law, that that alteration should not be at once as extensive as the circumstances of the case absolutely require.

The author complains of the defective manner in which medical evidence is received, and establishes by arguments which we need not quote, that that evidence only should be required or received which is delivered by competent witnesses. (p. 56.) He has, however, endeavoured to illustrate his views by two unfortunate examples. He has trusted to Foderé for his account of an English case which is totally misrepresented by that author. In the case of Fish (not Fischer) *v.* Palmer, Dr. Denman's opinion was properly rejected by the court, not in consequence of the testimony of three ignorant non-professional persons, although their evidence related to matters of *fact*, but in consequence of its being opposed to the opinions of Drs. Babington and Haighton, men equally eminent, and to the rules of common sense and equity. When Dr. Ray has read this case, we think he will be inclined to alter his opinion.* The reference to John Hunter's evidence in Donellan's case is still more unfortunate. This was a question in toxicology, with which that eminent surgeon confessed he was but little acquainted. We have yet to learn that eminence in one department of the profession is to secure implicit belief for opinions expressed on any subject.

In treating of mental diseases, the author commences with imbecility and its legal consequences. He gives the five degrees of this state proposed by Hoffbauer, and thinks that they are not entirely devoid of utility. The divisions in a practical view appear to us useless and absurd; since, before we can assign a particular case to its proper division, we must have attained all that medically or legally we require to know concerning the state of mind of the party. The case of Miss Bagster (p. 82) does not furnish us with a favorable specimen of the author's powers of analysis. He comes to the conclusion that the verdict was correct, and that this lady was really imbecile to a degree requiring legal interference. A full report of this case has already appeared in a contemporary journal, (Med. Gazette, vol. x. p. 519,) and we know of none which shows a more dangerous use to which the machinery of the law may be put. This young lady inherited a large fortune. Her education had been much neglected, but nevertheless she does not appear to have been treated as one of unsound mind, nor was any question of her sanity raised until she had contracted a marriage disapproved of by

* An abstract of it will be found in No. XVII. of this Journal, p. 45, (January, 1840.)

her mother; by which marriage the control of her property would be affected. Seven medical witnesses, summoned to support the commission, deposed that she was of unsound mind: on the other side, the counsel called no witnesses: but the commissioners themselves summoned two men of eminence, who deposed that her incompetency to manage her affairs arose, not from unsoundness of mind, but from ignorance. She was ignorant of arithmetic which she had not been taught, nor could she give satisfactory answers respecting the expenses of housekeeping; for in these matters she had never been instructed. She was, in the opinion of Dr. Morison, capable of instruction, and gave one strong proof of sanity, namely, that she was aware of the deficiencies under which she laboured.

It is probable that, besides ignorance, there was some degree of weakness of mind about her; yet, taking the whole case, we must attribute the verdict of unsoundness not so much to mental infirmity as to incapacity, from want of instruction, to manage a large fortune. But if every wealthy young lady whose education had been much neglected, had her sanity tested on the same points as Miss Bagster, we are certain that many who are now free agents would be placed under interdiction.

Dr. Ray's opinion of this case has probably been formed from his having seen only a short and imperfect abstract of the proceedings. We differ from him in thinking that her not being capable of taking care of herself or property was a proof of the mental deficiency being constitutional. Upon such weak grounds as these many young persons of both sexes might be immediately placed under restraint and their property surrendered to the cupidity of relatives. All the points in Miss Bagster's case which tended to show her sanity, the author fritters away under the possibility that they may have been exceptions to the rule. Thus her self-control and the concealment of her prominent deficiencies are not set down to the exercise of a sane mind, but to the exceptional case of an imbecile being sometimes capable of displaying this power. Her answers to the examiners, he admits, were pertinent and properly delivered; but still, he says, this is consistent with the first degree of imbecility of Hoffbauer. Her capacity to receive instruction is not looked upon in any other light than as an exception, since some imbeciles are also susceptible of education. Her acknowledgment of her deficiencies, and her expressed wish not to be asked questions on subjects which she had never been taught, he does not notice. We would, after this, simply ask the author whether, upon the principles by which he here professes to judge of mental capacity, a large portion of society might not be pronounced insane, and interdicted from all civil functions? We can never assent to the dangerous doctrine that the bare incompetency to manage affairs is to be taken as a test of unsoundness of mind, without the most satisfactory enquiry being made into the cause of that incompetency. It was never intended by the law that the ignorant and uneducated should be classed with the insane, or that the property of wealthy minors should be protected at the expense of their civil liberty.*

* Two other points in this case are worthy of fixing our attention. 1. It is generally held in law to be a proper rule to give the benefit of any doubt to the party affected by the proceedings. In this case, there must assuredly have been much doubt, for the only two independent medical witnesses in the case, i.e. those summoned by the commis-

The author devotes some space to the characters of *mania*, which he divides into intellectual and moral, and these again are each subdivided into general and partial. The subject is well treated and illustrated by numerous cases, most of which are borrowed from French and German writers. We must refer to the work itself (p. 208) for an excellent summary of the main features of homicidal insanity,—the principal of which he considers to be an *irresistible motiveless impulse to destroy life*—as also for the means of forming a diagnosis between it and homicide, as it is perpetrated by a sane criminal. The circumstances connected with the civil disabilities of those labouring under the various forms of mania and under dementia are treated more at length than in Dr. Pagan's work, and the illustrations, chiefly from our ecclesiastical reports, are numerous and appositely selected. The chapters on the legal consequences of delirium and on lucid intervals are worthy of attention; and we think the author has acted wisely in separating the last subject from the general description of insanity. If we do not extract any of the remarks made on these points, it is simply because we have already fully discussed the questions to which they refer. We pass over the chapters on feigned insanity, suicide, and somnambulism, and come to those "of the Effect of Insanity on Evidence," and the "Legal Consequences of Drunkenness." These subjects are only cursorily noticed by Dr. Pagan.

It has often become a serious question in enquiries before coroners, whether a person labouring under insanity is competent to give evidence as a witness. When a death takes place in a lunatic asylum, and there is an allegation of ill-treatment, the truth of the charge can sometimes be proved or disproved only by evidence of this description. In confirmed mania or in dementia, it is impossible to admit the testimony of parties;—the slightest examination would at once show that the mind was incapable of combining or arranging in any consistent order the impressions which had been received through the senses. As a general principle, the insane, whatever be the form of the malady, are disqualified by law from appearing as witnesses in courts of justice, "their incompetency being *inferred* from their mental unsoundness." (p. 361.) It therefore lies with him who produces such persons as witnesses to show that there is reasonable ground for trusting to their statements. In partial insanity, competency is occasionally allowed, although it requires a great knowledge of the case and extreme caution to measure the witness's credibility. One serious objection to all such evidence is, that the mental impairment, even when slight, is often accompanied by a total indifference to truth or falsehood, and there is a tendency in the lunatic to misrepresent or exaggerate every circumstance which comes before them. We cannot agree with Dr. Ray that when the matter on which the lunatic testifies "is remote from the insane delusion which he entertains, and cannot very obviously come within the circle of its influence, it sioners, Dr. Haslam and Dr. Morison, two men of great experience, thought that there was no ground for legal interdiction. Two jurors out of twenty-two thought the same, yet the benefit of the doubt was not given to this young lady. 2. All agree that if this case was one of insanity at all, it was one of imbecility. Now this is a condition which exists from birth or early infancy: the evidence did not show that there had been any change of character in Miss Bagster; on the contrary, it appears to us to establish that she was the same throughout. Yet singularly enough, the verdict of the jury dated her insanity only *two years* prior to the commission.

would be wrong to reject his testimony on the score of incompetency," (364) because it is not possible for us to determine the exact degree in which the witness's statements may be influenced by his delusion, or the relation between them. A better rule is this: when their statements are borne out by those of other witnesses and corroborated by circumstances, then we may trust to them. A jury would, however, always be guided by the result of their evidence: thus if it affected the life of a prisoner charged with a capital crime, notwithstanding its corroboration by circumstances, it would probably be rejected. A case establishing this is related by the author (p. 365).

Some doubt may arise as to the competency to give evidence, in aged persons labouring under incipient dementia, and such evidence is often required in reference to dates of birth, the existence of local customs, and the execution of wills and deeds. There is no fixed rule as to the admission of this kind of evidence, which sometimes involves to a great extent the rights of persons and the disposition of property. It has been remarked, however, of such persons, that they may have a distinct recollection of events which took place many years before, while their memory is not to be trusted with regard to recent occurrences. If an aged witness can remember other transactions about the same date as that on which he has to give evidence, then we have a strong presumption in favour of his competency.

In a previous part of this article we have slightly touched upon the effects of drunkenness, on the difference between mania and delirium tremens, and on the legal responsibility of persons labouring under the last-mentioned disease. It is now our purpose to complete this subject by considering the legal consequences of acts committed by persons while in a state of drunkenness. Passing over Hoffbauer's three stages, we may remark that jurists and legislators have differed widely as to the degree to which drunkards should be made responsible for their acts. When the mind is weakened by habitual drunkenness, then the law infers irresponsibility, unless it be made plainly to appear that the individual was, at the time of the act, endowed with consciousness and reason; but the question at present is, how far an ordinary fit of drunkenness creates irresponsibility.

Any deed or agreement made by a party while drunk is not invalidated by our law, except in the case where the intoxication has proceeded so far as to deprive him of all consciousness of what he is doing; and a court of justice will not interfere, unless the drunkenness were the result of collusion by others for the purposes of fraud. Thus if three or four persons were to combine to intoxicate another, and to marry him while in that state, a court would not hesitate to annul such a marriage on clear proof of the fact. The validity of a will or deed would rest upon similar principles. It is easy to perceive by this that the law makes two states of drunkenness: one where it has proceeded to but a slight extent, and where it is considered there is still a power of rational consent; another where it has proceeded so far that the individual has no consciousness of the transaction, and, therefore, can give no rational consent. The proof of this last state would vitiate all the acts of the party. Some have held that the proof of drunkenness, under any circumstances, should vitiate all acts. We must certainly look to the distinction drawn by the law of the existence of a power of rational consent in the first stage, in the light of a punishment on the party, since, although we cannot deny consciousness, we must consider that the judgment is weakened under the excitement produced by intoxicating drinks. A man thus situated is not in a condition to judge clearly of the rationality of his acts.

Is the evidence of a drunken person, relative to acts committed on him while in a state of drunkenness, receivable in a court of law? This is a question very often raised, since persons, whether intoxicated by their own voluntary act or designedly by others, are often subject to robbery. In general, all such evidence is treated with the greatest suspicion, and unless corroborated by the testimony of others, or by circumstances, it is rejected. When the drunkenness is designedly produced by others, a very slight corroboration will often suffice to render it receivable. In this distinction we again see that voluntary drunkenness is visited as a punishment on the party.

A man is held responsible by law for a robbery committed by him while in a state of drunkenness; and a curious case has just been decided (March, 1840,) on the Norfolk circuit, in which one drunken man was charged with highway robbery on another man who was also drunk. The prosecutor admitted that he was not sober, but denied that he was drunk! The jury rejected his evidence, because it was entirely unsupported by circumstances, and acquitted the prisoner.

A confession made by a man while in a state of drunkenness is legally admissible as evidence against him or others, if corroborated by circumstances. This is a most important point in regard to the responsibility of drunkards; and a case which has recently occurred leaves the English law undoubted upon this point. A man confessed while drunk that he had committed a robbery and murder, which had taken place some time before, but of which he had not been suspected. He mentioned the part of his cottage where the property of the murdered person had been concealed by him, and the whole of the circumstances of the murder. The property was found as he had described, and the case was clearly brought home to him, chiefly by collateral evidence from his own confession. He was convicted.

When homicide is committed by a man in a state of drunkenness, this is held to be no excuse for the crime; on the contrary, from the dictum of Lord Coke, the English law would regard it as matter of aggravation. The common law on the subject, as expounded by that great authority, is that "a drunkard, who is *voluntarius dæmon,* hath no privilege thereby, whatever ill or hurt he doeth, his drunkenness doth aggravate it." Practically speaking, in the present day, while drunkenness is not allowed by our judges as an exculpatory plea, it is not regarded as a source of aggravation. In no case is it, if voluntary, admitted as a ground of irresponsibility, even although the party might not have contemplated the crime when sober. Indeed, some judges have taken so rigorous a view of the case, that they have not even admitted the plea of exculpation when the crime was committed in a state of frenzy from *habitual drunkenness.* This, however, is not the general interpretation of the law, although, as we have before remarked, we do not see why, if responsibility is diminished in one case it should not be in the other; since, morally speaking, there can be no difference in the criminality of the act. Although it would be a dangerous principle to exempt men from responsibility for crimes committed in a state of intoxication, yet, as the reasoning faculties are weakened, and the power of self-control is much impaired, it seems harsh in any law to punish such persons in the same way as criminals who enjoy their faculties, and have the power of controlling their feelings. The late Sir James Mackintosh judiciously remarked, in relation to this question, that as the example of punishment does not influence the man who is drunk any more than one who is mad, it is plain that to hang a man for what he does under such circumstances is to make drunkenness, when followed by an accidental consequence, a capital offence. His execution will not deter drunkards from murder; if it operate in any way, it can only deter men who are sober from drunkenness. The following instance occurred to him while he was officiating as a judge in India. An Irish artillery-man, in a violent fit of drunkenness, wrested a sword from the hands of a comrade, ran with it two or three miles on the road, and at last killed a poor old, unarmed, and unoffending seapoy of police. There was nothing to extenuate the crime, except the drunkenness; the prisoner was respited. This case so perfectly resembles one of homicidal mania, in the want of premeditation, precaution, concealment, and the absence of motive, that if ever that form of insanity were allowed to exempt from capital punishment, there was an equally strong reason for exemption in this instance. We cannot assent to the justice of the view, that the drunkenness being a voluntary act, and the homicidal mania having an involuntary origin, the cases are different; because, in the first place, this is virtually equal to visiting drunkenness with capital punishment, and, secondly, it is looking to the cause and not to the fact of the derangement of the mental powers. Supposing, as Dr. Ray has suggested, an homicidal monomaniac has brought on his destructive impulse by a voluntary attendance at exciting religious or political meetings, would the law visit a crime, committed by him in this state, with greater severity than that committed by another whose homicidal impulse spontaneously attacked him? If drunkenness is never to be admitted as an exculpatory plea on such a ground, then, in common justice, wherever insanity is pleaded for crime, the court ought, before granting it, to be satisfied that the individual has not brought on or aggravated his condition by his original misconduct. Such a doctrine is wholly untenable; for in the eye of the law exemption exists where there is a loss of power to distinguish between right and wrong, and in the view of the psychologist that exemption should be extended to all cases where there is a manifest loss of power of self-control. Neither in a moral nor in an equitable light is there any reason for distinguishing crimes committed during drunkenness from those perpetrated in the state of insanity; the plea of irresponsibility should be governed by the same principles in the two cases, and imprisonment substituted for capital punishment.

We do not know that we can add to these remarks by quoting cases which, unhappily in England, are too frequent; but we could point to several in which the severe penalty of death has, in our opinion, been most unjustly inflicted. We sincerely hope that the time is not far distant when a more enlightened view of this question will be taken, when it will not be allowed to rest on the individual opinions of judges, but will be fixed by rules of justice and humanity.

There is a class of cases differing slightly from those above considered, in which persons have sustained injuries to the head, as often happens with soldiers and sailors, where drunkenness, even when existing to a slight extent, produces sometimes temporary insanity, and leaves the mind in possession of its habitual sanity when the drunken fit is over. The law does not appear to draw any distinction between this state and ordinary drunkenness, although juries occasionally show, by their verdicts, that some difference ought to be made. Such persons certainly ought not to undergo the same punishment as sane criminals, unless the crime be accompanied by many circumstances of aggravation, and the plea rest rather upon suspicion than upon proof.

The work closes with a chapter on Interdiction and Restraint, which are not necessarily dependent on each other—interdiction being confined to the deprivation of the person of his civil rights. We shall not follow the author in his account of the circumstances which would justify interdiction in the various forms of insanity; but he appears to us to have unconsciously involved himself in some contradictions. At p. 412 we find him asserting that the accurate test of capacity, in a person labouring under imbecility, is the manner in which he has *already* managed his affairs; but this test is wholly inapplicable to those instances in which, from want of instruction, neglect, or the infancy of the party, he has never learned the method of managing property. The dishonest guardians of wealthy minors might take advantage of this principle to prevent their wards from ever acquiring a control over their estates. He complains of the facility with which interdiction is granted in Great Britain; but forgetting that he himself has justified this practice in the case of Miss Bagster—in our view one of the most disgraceful in modern times—we find him saying:

"One finds it difficult to believe on what slight grounds interdiction is there (in Great Britain) every day procured—a measure that, with the ostensible purpose of protecting the interests of the insane party, is too often in reality designed to promote the selfish views of relatives and friends. A kind and degree of mental impairment that has never obscured the patient's knowledge of his relative situation, never altered his disposition to be kind and useful to those around him, never weakened his enjoyment of social pleasures, and never affected his capacity to manage his concerns with his usual prudence, has been repeatedly deemed a sufficient reason for depriving him of the use and enjoyment of his own property, and subjecting him to all the disabilities which the law can impose." (*Ray*, p. 414.)

He justifies this censure by a reference to the celebrated case of Davies, the teadealer, and pronounces him to have been sane because he could manage his affairs—i. e. buy and sell. In our opinion there were far greater evidences of insanity about the general conduct and habits of this gentleman—to which in other parts of the treatise (p. 81) Dr. Ray directs us to look for proofs of imbecility—than about Miss Bagster; and, although it is admitted that Mr. Davies bought and sold and managed his affairs with ordinary care, we cannot understand why this "commercial test" should be applied to any young lady whose age, position in society, and future prospects must have made such a mode of examination a fruitless mockery. Yet it was chiefly upon this ground she was interdicted; and in spite of the above judgment, we find Dr. Ray contending in her case, without regard to her youth or want of education, that the power of self-control, the appropriate answers returned to questions, and the capacity to receive instruction, were no evidences of sanity, but conditions of the mental faculties occasionally met with in imbeciles! We agree with him in condemning the English practice; but there can be little hope of improvement so long as experienced physicians like himself are led to advocate interdiction upon such weak and untenable grounds. The severe censure which he has passed upon the medical witnesses in Davies's case (p. 418) must, to a certain extent, recoil upon himself.

Notwithstanding there are many points in which we have taken leave to differ from Dr. Ray, we must do him the justice to assert that he has on the whole well treated his subject. His views are generally sound and practical, and display considerable acquaintance with some of the most obscure problems of psychology. His language is occasionally rather vehement,* but we believe this has arisen from a sincere wish to bring the jurisprudence of England and America, relative to the insane, up to a humane and scientific standard.

3. The third work on our list, by M. Cazauvieilh, professes to treat of *Suicide, Mental Alienation,* and *Criminal Offences;* but it embraces such a variety of subjects, and these are so loosely connected with each other, that we are quite at a loss how to characterize it. The medical jurisprudence of perforations of the stomach, drowning, hanging, and strangulation, are successively discussed; and to these we find appended the Statistics of Suicide, with the author's views of the criminal responsibility of the insane. Many cases of suicide are detailed, with an account of the post-mortem appearances, internally and externally. There is much industry displayed in the collection of these, but we have some difficulty in finding any facts which throw light upon the subject; for most of the appearances are evidently wholly unconnected with the state of suicide. The author has adopted the idea that *induration* of the cerebral substance is one of the most marked morbid changes, but varying in degree, according to whether the suicidal malady has been of an acute or chronic character. Whether this induration is a primary or secondary consequence of the malady he is unable to say; but he thinks it useless to attempt to dive further into the nature of the proximate cause. (p. 187.) Unfortunately for this hypothesis, out of seventeen inspections, induration was met with only in seven. In four cases out of the whole number the brain was not examined; but this would leave six cases in which the alleged morbid character was wanting. The author accounts for this objection to his view, by supposing either that the suicidal disposition had not existed long enough to harden the encephalon, or the brain was affected sympathetically by the other viscera, which were in a state of chronic disease. We prefer considering that the connexion between cerebral induration and a suicidal disposition was not made out.

He finds statistically that suicides underwent an annual increase in France from 1827 to 1835, except in 1830. The deficiency in the year 1830 he accounts for by supposing either that men's minds were directed by political events to other subjects or that there was some mistake in

* Vide pp. 48, 25, and elsewhere.

the tables. The first reason is altogether contrary to experience, since, as Dr. Cazauvieilh ought to know, suicides and cases of insanity are generally much increased by political changes. The yearly number of suicides in France amounts to 2000. (p. 3.)

Most of the author's statistical details are of a local character, so that they would only interest a French reader.

The last portion of the volume is devoted to the subject of homicidal madness. In this the author chiefly follows Georget. The remarks contain nothing new, but on the whole, the circumstances which should regulate responsibility are fairly detailed. There is a certain diffuseness of style, with a disposition to fine writing, which often renders the author's meaning obscure. The work is closed by an account of the treatment of insanity, with a tendency to suicide; but his ideas on this subject seem to us vague and unfitted for practical application.

4. The object of the *Barrister*, in his Letter on the *Law of Lunacy*, is to show that the present system of consigning alleged lunatics to asylums by medical certificates is dangerous to the liberty of the subject. But he appears to us to take a most narrow and illiberal view of the question. Indeed, we may consider his pamphlet as a direct attack on the members of the medical profession. Thus at p. 9 we find him insinuating that some surgeons from their poverty might be bought over to sign "the fatal document" by the bribes of avaricious relatives. Although abuses have taken place, we do not believe there ever existed any ground for such an imputation as this; and we are quite satisfied that in the present day, if no other principle restrained a man from granting a certificate improperly, the certainty of detection would deter him. He proposes that instead of this power being vested in medical practitioners it should be intrusted to the decision of a jury of twelve men, under the superintendence of a county coroner, to act as judge, the case being supported and defended by the technical ability of any number of counsel. If such a scheme as this were brought into operation, we fear there would be no end to litigation and expense. One half of the alleged lunatic's estate would go to settle whether he should be confined, and the other half, under a commission, to determine whether or not he was a fit subject for interdiction. While we admit with the "barrister" that the present system of allowing men unacquainted with the subject of insanity to sign these certificates is a highly unwarrantable practice, we think the whole of the grievance would be removed by simply restricting the power to those who had made this subject a special study.

The author thinks that commissions "*de lunatico inquirendo*," might be continued on their present footing, as his scheme would not interfere with them. This satisfies us that he has taken a very partial view of the law of lunacy. In each of these commissions, we find officiating generally three commissioners, from *nineteen* to *twenty-three* jurors, the sheriff and his officers, counsel on both sides, an unlimited number of witnesses, while the sittings are often protracted to twelve or fourteen days—and all this display of legal machinery is at the expense of the lunatic or his friends. The cost of such a commission often makes the most serious inroad into a lunatic's estate; and, after all, are the results of these protracted and expensive proceedings proportionately satisfactory? We would refer for an answer to this question to the conflicting verdicts in the cases of Mr. Davies, Miss Bagster, and many others.

A little reflection will show, that the question for the commission to solve might be decided with as much certainty, and with infinitely less expense to the parties, by doing away with one half of the legal functionaries, and reducing the number of the jury and witnesses within a reasonable limit. These commissions are only suited to the wealthy classes; and we often find, the larger the fortune at stake, the longer the proceedings are protracted, so that they afford little or no protection to the middling and poorer ranks of society. It may be plausibly urged, that in a question affecting the disposition of a large property, delay is necessary for a safe decision; but we deny the inference in this case, and point to the numerous instances in our courts, where *one* judge and *twelve* jurors, at infinitely less expense, are deemed able, not merely to decide questions involving to a far greater extent the rights of persons, but to determine, sometimes in a few minutes, whether an alleged lunatic is responsible to the law for a crime which may affect his life! Now it is certain that injustice must be done to individuals in one of these cases, and we incline to think that the injustice is on the side of these expensive and protracted commissions.

With such facts before us, we must suppose that there was either a want of knowledge or a want of candour on the part of the "barrister" when addressing the lord chancellor on the "present state of the law of lunacy," in not denouncing these commissions as producing grievances of much greater magnitude than that of which he complains. If the liberty of the subject should ever be infringed, as he supposes, there is an ample remedy; but, so far as we know, there are no means of preventing the enormous pecuniary loss which a lunatic must sustain in order to receive the protection of the law!

5. Nearly simultaneously with the English and American works already noticed, we find appearing in France the more elaborate treatise of M. Marc on the same subject. We shall take advantage of this circumstance to compare the views of this distinguished writer on the medical jurisprudence of insanity with those detailed in the preceding part of this article. At the same time, with a due regard to the space which our pages will afford, we shall rather make selections capable of furnishing additional illustration than go over the same ground again. As our journal is chiefly addressed to English readers, we have preferred taking English works for our guides, since it is to these that the British practitioner will chiefly look for practical assistance in these difficult enquiries.*

The treatise, as we have already remarked, is on a much more extended scale than those of Drs. Pagan and Ray, and the subjects are treated in a somewhat different order. In the first volume we find discussed: medical competency—moral liberty—hallucinations and illu-

* It is with regret we find, from a note attached to the preface, that the author died suddenly of apoplexy on the 12th January last, immediately after completing the work. A short account of his labours is appended to it, which show that medical jurisprudence is much indebted to him.

sions—general remarks on insanity, and the medico-legal relations of idiocy, imbecility, and mania. It will be seen by this that the author adopts the divisions of Pinel and Esquirol. In the second volume the subjects of homicidal and suicidal madness, erotomania, demonomania, kleptomania (stealing), pyromania (incendiarism), dementia, temporary insanity, drunkenness (dipsomania), and, lastly, the civil relations of insanity are successively considered. M. Marc has not been sparing of illustrations, some of which he borrows from Esquirol and other sources, but many of them are founded on personal observation; among these last, we recognize some as having already appeared in detached essays, or in the numbers of the Annales d'Hygiène. The author has adopted in this respect a somewhat novel plan, namely, in many instances that of inserting at full length the whole of the legal proceedings, so that the cases thereby make up a large portion of the bulk of these volumes. In his preface he apologizes for this, and thinks that had he merely given abstracts of them, their authenticity would have been diminished. We do not quite agree with this view, since much must be necessarily published which can throw no light on the medical part of a case, and which will only serve to swell out a volume to an unwieldy size. Had M. Marc pursued the usual plan, we are certain that his name would have guaranteed the authenticity of his cases.

The question of "medical competency" will not detain us. The author expends a great deal of argument in refuting the views of those who wish to place insanity under the jurisdiction of metaphysicians and lawyers; and he shows a praiseworthy zeal in insisting that well-informed members of the medical profession alone are fitted to examine and report upon all cases of mental alienation. At the same time he admits that the subject is one which should certainly claim the attention of jurists, for whether they act as accusers, judges, or defenders, they will find that a knowledge of it will materially aid their judgment, and enable them to perform their duties more efficiently.

Among other cases to illustrate the absolute necessity for medical interference, he brings forward that of Bernard Schimaidzig, who was tried for the murder of his wife, but acquitted on the ground that the act had been performed involuntarily, and while he was labouring under a delusion in a half-sleeping and half-waking state. (p. 56.) The investigation required to determine the responsibility of this man rested purely upon medical, not on legal or metaphysical principles.

"No case," observes the author, "offers deeper interest than that in which one is compelled to choose between the two extremes, of *entire irresponsibility* or the *greatest criminality*; no case more complex, or standing in greater need of philosophical illustration, than where the act is certain, but its moral consequences are disputable; where the ordinary means of proof, by witnesses, reports, or confessions, avail nothing, but we are obliged to have recourse to a species of artificial proof, formed of many minute circumstances, general principles, observations, and even probabilities." (*Marc*, p. 66.)

Our impression is that this "argumentum ad judicium" has become in the present day supererogatory. We have never heard the shadow of an argument for the subject of insanity being taken out of the hands of the profession; and we are quite certain if the advocates of this view were temporarily successful in their efforts, the mischief which would result would soon cause the privilege to revert to its members.

The chapter on *moral liberty* is deeply interesting. In the author's opinion, the ground for exempting the insane from legal responsibility is, that their acts are *involuntary*. This leads him to speak of volition, which he defines to be "a faculty producing, directing, preventing, or modifying the physical and moral acts which are submitted to it." Again, "This normal state of the will constitutes moral liberty or free judgment. The being in whom this faculty is diseased becomes thereby incapable of controlling his actions, and may be classed among the insane." (p. 85.) The essence of insanity, according to this, would consist in what some physiologists have termed a diseased or paralysed volition. The author is evidently aware that this doctrine is not free from objection, for we find him shortly afterwards admitting that it would become a matter of great difficulty to apply it as a test of criminal responsibility. (p. 121.) Volition is not necessarily or equally impaired in all cases of insanity; while, on the other hand, it is often considerably impaired under the effects of extreme passion. Besides, in what difficult and imperceptible degrees does this power of the will exist among the sane? The chapter is concluded with an account of the various passions, and of the circumstances which would lead us to determine whether a particular act had been committed with or without the presence of moral liberty in the individual. The author's views here are so generalized, that we find it impossible to condense them. We must therefore refer our readers to the work itself.

*Hallucinations* and *illusions* are often confounded by those who treat of insanity.

"Hallucinations are those sensations which are supposed by the patient to be produced by external impressions, although no material object acts upon the senses. Illusions, on the other hand, are the results of a material action on the perceptive faculty, although the object is falsely perceived. When a man fancies he hears voices speaking to him while there is the most profound silence, he labours under a hallucination. When another imagines that his ordinary food has an earthy or metallic taste, this is an illusion." (*Marc*, p. 174.)

Although hallucinations may exist without any decided mental disorder, besides that requisite for their production, yet they may give rise to conceptions bordering on insanity. These last may also excite hallucinations without our being able to determine the priority of either state. Hallucinations and illusions are very frequently combined in insanity. (p. 181.) In two thirds of all cases the hallucinations are confined to the sense of hearing. The sense of vision is not so often affected, if we except the state of sanity in which, if there be hallucinations, this is the sense through which they chiefly appear to operate. Considering them as distinct from illusions, to which the eye more than the other senses is subject, there are few persons who have not experienced hallucinations of vision. In weak and superstitious minds, these false images have been mistaken for real apparitions. (p. 188.)

It must not be supposed that in insanity these hallucinations are confined to a single sense. The eye and the ear are often simultaneously affected. Illusions are not merely produced by the external senses; they often accompany internal sensations, and the character of the delusion in the insane is considerably modified by them. Illusions are sometimes met with in the sane; but when they are habitual, more or less persistent, and are not under the control of the reflective faculties, they may produce or complicate the state of insanity, giving rise to the most wild and unconnected ideas. Although hallucinations and illusions are main features of insanity, they are rarely met with in cases of idiocy and imbecility, sometimes in dementia, most frequently, however, in monomania and mania, especially in the paroxysms of the last-mentioned state.

A good deal has been already said respecting the connexion of the acts of lunatics with the delusions under which they labour; and we have endeavoured to show, that to require it to be proved as a necessary ground of irresponsibility, is absurd. We cannot, in all instances, determine any such connexion from the language or conduct of the insane. M. Marc mentions the case of a man who for many years had been in the habit of licking the walls of the apartment with his tongue, until he had actually worn away the plaster. No one could imagine what was the cause of this perseverance in so painful and disgusting a habit, until one day in the author's presence he confessed, that he tasted and smelt the most delicious fruit on the walls. The discovery of this connexion must be often the result of pure accident; but there is no doubt that, wherever the acts of a lunatic are very unusual and strange, they depend on the presence of some hallucination or illusion. (p. 195.)

In treating of the general forms of insanity, the author commences with idiocy and imbecility. He very properly condemns Hoffbauer's divisions of the latter state as hypothetical subtleties, totally inapplicable to the practice of legal medicine, an opinion fully justified by several cases related in the subsequent pages. Idiocy being a congenital affection, and imbecility showing itself only in the first periods of life, neither state can appear suddenly; a fact important to bear in mind in cases of feigned insanity. Pinel, it is true, speaks of these conditions being suddenly produced by violent emotions; but his description of them coincides with what is now more generally termed dementia.

M. Marc laments, with Drs. Pagan and Ray, that jurists and legislators have shown such violent opposition to the admission of homicidal monomania as an exculpatory plea, notwithstanding the overwhelming evidence of its existence accumulated by experienced physicians of all countries. But he says the objections to this doctrine do not arise simply from prejudice on the part of magistrates: "In applying their views with too little reserve, physicians have themselves contributed to retard their reception. It is with new doctrines as with new discoveries, however true or important they may be, no greater injury can be done to them than by the endeavouring to carry them too far. Under these circumstances, one (false) application of them will diminish their value, and create distrust of their reality." (p. 229.)

There can be no doubt that to come forward with a plea of insanity for every criminal, must lead to the rejection of that plea, where it is deservedly made. In this respect, the writings of Georget have done much mischief; for it has by a reaction, thrown the whole doctrine into disrepute with French jurists. We may observe here, that some practitioners, with more humanity than discretion, are in this country occasionally betrayed into a similar fault; for evidence has been given of the existence of insanity in criminals where there was not the least reason to suspect it, and there was no circumstance to mitigate the crime.

But there is another point on which medical witnesses are not sufficiently cautious, and that is, they make themselves advocates instead of witnesses; in which case they necessarily incur the risk of taking upon themselves the responsibility of a bad cause. Marc's observations apply more to criminal cases in France; but it is chiefly in civil suits in this country that we discover this most degrading bias in medical evidence. The author is right in contending that the witness, in giving his opinion on the state of mind of a party, "should place himself between the accusation and defence; he should wholly forget whether his opinion was demanded by the prosecutor or the prosecuted." (p. 231.) This is expecting almost too much from human nature; but it is nevertheless a desirable standard for the witness to endeavour to attain. We have already expressed our opinion strongly on this subject. The evil must continue in all the civil proceedings of this country, so long as parties are allowed to *retain* their own medical witnesses. Thus we see that in all cases of difficulty on commissions, medical opinions neutralize each other.

M. Marc thinks that, on the whole, French jurists are beginning to take a more liberal view of this question. Medical witnesses of experience are now frequently consulted, and greater care bestowed on the examination of the case (p. 238.) He admits two kinds of homicidal monomania: the one "*instinctive*," the other "*reasoning*." "The first incites the person, owing to a diseased volition, to instinctive automatic acts, not preceded by any reasoning on the subject; the second induces acts which are the manifest result of an association of ideas." (p. 244.) The first or instinctive state, in a medico-legal view, is much more difficult to establish than the other. Cases illustrative of these conditions are quoted from Mende.

Dementia, it is well known, may appear suddenly from violent emotions. The following is a singular illustration:

"During the republic, an artilleryman proposed to the Council of Public Safety a new species of cannon, which was to have the most deadly effects in war. A day was appointed for the trial of it at Mendon, and Robespierre wrote a letter to the inventor in such flattering language, that the poor man became motionless on reading it, and was conveyed to the Bicêtre in a state of complete idiocy (dementia)." (*Marc*, p. 269.)

In dementia the powers of the mind are destroyed, and there is something fearful in the thought that what it may have taken many years to build up may be thus annihilated in a moment by a strong emotion.

The subject of feigned insanity requires no further notice, since the means proposed by the author for its detection are the same as those which we have already detailed. A difficulty might occur in distinguishing these cases, from the fact that the forms of insanity are often mixed; but, says M. Marc, "these complications are only exceptions, and we may lay down the rule that when insanity is real, its characters do not differ materially from those of each of its principal varieties, with which we ought always to compare the symptoms met with in a supposed case."

(p. 276.) The causes of insanity, and the circumstances which modify its accession and duration, are amply detailed and illustrated. We may say the same also of the diagnosis and special characters of its various forms, as well as of the circumstances involving responsibility in the deaf and dumb. Some of the cases in illustration are unusually prolix, that of Gilbert, for example, with which the volume closes, occupies no less than forty-eight pages! We think this, with others, would have admitted of judicious abbreviation.

The second volume commences with an account of the characters of homicidal and suicidal madness, the materials for which, the author admits, he has chiefly borrowed from Esquirol. The illustrations are numerous: among them we find the case of Henriette Cornier, the publication of which, some years since, first established M. Marc's reputation as a medical jurist. We find nothing in these chapters to add to what we have elsewhere said on these subjects. In regard to suicide, we learn that the legal customs of France and England are different. In France the enquiry is limited to whether the act has really been one of suicide or not. In England it is thought necessary, in addition, to enquire into the state of mind of the deceased, in order, if a verdict of insanity should not be returned, to punish his surviving relations for the act by a forfeiture of his property! This enquiry is a mere matter of form, for neither coroners nor their juries are fitted, in the absence of good medical evidence, to pronounce a verdict on such a point. It is remarkable, however, that in cases of unquestionable suicidal monomania, English juries generally return a verdict of *felo de se* (Steinberg's case); and where there is every ground for supposing that the individual possessed reason until the last, they commonly pronounce a verdict of insanity. The author, although he avoids the question, does not regard suicide as a necessary proof of insanity. (p. 169.) The previous state of the individual—the method by which he has destroyed himself—and the motives that led him to it, are circumstances which here require consideration. We fully agree with him in this opinion, and think that if suicide is always to be taken as evidence of insanity, parricide and infanticide should be regarded in the same light. This would be the doctrine of those who contend that all crime is the result of some kind of insanity.

M. Marc proposes the term Aidoiomania (*αἰδοῖον*, pudenda,) for Erotomania, a term used by Esquirol to designate those states of mental disorder connected with sexual propensities. This chapter, together with that on Demonomania, we pass over, as offering nothing requiring further notice. The subjects of Kleptomania and Pyromania are treated at some length. It has been remarked that a propensity to steal has often existed in females advanced in pregnancy, the motive being the mere wish of possession. Pregnancy, he thinks, should be a good exculpatory plea, where a well-educated person of strict moral conduct steals some unimportant useless article, of no value compared with her fortune. At any rate, the rejection of it would be great injustice on the old axiom: *Summum jus, summa injuria.* (p. 274.) Cases of this kind occasionally present themselves at our police courts, where this motiveless impulse for theft is pleaded by persons moving in highly respectable society. We are not aware that English practitioners have at all connected it with the pregnant state. The rules for forming an opinion in these cases have already been alluded to.

Pyromania has been chiefly observed among young females about the age of puberty, in whom the menstrual discharge has taken place with difficulty or great irregularity. The points to attend to in such cases, where no criminal motive for the act is apparent, would be: 1, the age, varying from the twelfth to the twentieth year; 2, the presence of symptoms indicative of an irregular bodily development; 3, of those indicative of irregular sexual development (for by this the brain may become sympathetically affected); 4, any marks of disorder in the cerebral or nervous system, indicated by change in the moral character. (p. 378.) Nevertheless the want of positive evidence of mental disturbance, or the proof of sanity in the individual, must not mislead the physician, since moral liberty may be lost without a disturbance of the reasoning faculty. (p. 382.) Motives may be discovered, among which in these young persons we find hatred, revenge, mischief, discontent, nostalgia, or even the desire to see a fire on a large scale!—any one of these may exalt the pyromaniacal feeling and lead to incendiarism. The author is sensible that he is here treading on dangerous ground; he seems to us to want independent views on this subject; for much of the chapter is occupied with the different opinions of German psychologists, on the circumstances which should create irresponsibility. A plea of this kind, unless admitted with the greatest caution, might become a means for withdrawing real criminals from all legal control.

An interesting chapter follows on "*Imitative Monomania.*" Monomania, either "reasoning" or "instinctive," may be propagated by imitation. The first, being the result of erroneous ideas, is easily transmitted; these ideas inflame weak and enthusiastic minds, and thus we may account for the spread of monomania, whether connected with religious or political notions. Numberless examples of this kind might be quoted. (p. 402.) In our own country we might point to the case of Thom, whose conduct led to the destruction of his own and many other lives, a few years since, in the neighbourhood of Canterbury. The religious delusion which emanated from this lunatic raised up many ignorant and uneducated followers, who devoted their lives for him. The histories of all countries and ages abound with such examples. Many instances of the diffusion of a political monomania might also be cited. Instinctive monomania, more difficult to explain in its origin, but more easy to recognize than the former condition, is also transmissible by imitation. We must refer to the work for many examples of this kind.

The transmissibility of suicidal monomania by imitation is so well known to psychologists, as almost to justify the application of the terms "epidemic or contagious" to this state. It is more especially observed, where the facts connected with the death are such as to create much notoriety, from the means of destruction being of an extraordinary nature. Very soon after one suicide took place by precipitation from the monument of London, another was committed; and the same has been remarked in Paris. M. Marc alludes to a suicidal club in Paris, consisting of twelve persons, who put themselves to death by ballot, one by one, yearly. (p. 422.) Well may we consider that the vagaries of the human mind are incomprehensible!

This principle of imitation may be seen in other cases: thus one act of incendiarism often leads to others, as has been unfortunately illustrated in the agricultural districts of England; but we believe that there may be a criminal as well as a monomaniacal imitation, and in these cases experience has shown there is no check so effectual as the rigorous administration of the law.

Under the head of "*temporary insanity*" the author treats not only of those mental disorders which come on suddenly and speedily disappear, but also those states of insanity which are accompanied by lucid intervals and regular or irregular intermissions. A lucid interval is commonly defined to be a temporary cessation of the insanity, while a remission is a mere abatement of the symptoms. Acts performed in the first state may be valid, if there is clear proof of the fact; acts performed during a remission are invalid. A lucid interval may last for a few minutes only, or it may be protracted for days, weeks, months, and even years. It may come on suddenly or slowly. In a medico-legal view, its alleged existence must always be looked on with suspicion where the interval has been short. The rules for determining this state have been already described. In criminal jurisprudence, M. Marc recommends that we should judge of the existence of a lucid interval by comparing the nature of any act performed with the motives which have led to its performance: whether these motives are proportioned to its gravity; and whether the act can be connected with any delusion in the mind of the supposed lunatic. (p. 500.) According to the quoted opinion of the Chancellor D'Aguesseau, the proof required to establish a lucid interval is much the same in France as it is in England.

Epileptics are subject to fits of temporary insanity, under which they may be guilty of acts, that may create a serious question of their legal responsibility. *Epilepsy* has been generally observed to lead sooner or later to insanity. The maniacal fury of epileptics, when once excited, is formidable, and has often produced the most fatal consequences. The older the fits of epilepsy, the more frequent and more violent they become, and the more likely are the intellectual faculties to be impaired. (p. 524.) The sooner the act committed has supervened on a fit of epilepsy, the greater the reason is there to suppose that it has depended on some disturbance of the mind. The responsibility of epileptics, a question often omitted by writers on the legal relations of insanity, requires much attention on the part of a medical jurist. M. Marc has furnished many cases which serve well to illustrate the difficulty of the subject.

Another form of temporary insanity is *drunkenness*. The author here confines himself to the consideration of that state in which the mental faculties are only temporarily impaired by vinous liquids. We gather from his remarks that the law of France is very similar to that of England, in relation to the criminal responsibility of drunkards. It is nowhere mentioned as an exculpatory circumstance, and the question of responsibility is made to rest rather upon drunkenness being a voluntary act than on the effects of intemperance on the mind. M. Marc lays some stress upon whether or not the crime committed in this state has been premeditated while the individual was sober; but we think this would be an unsafe rule to follow, unless it was proved that the accused had purposely taken intoxicating liquids, to excite himself to the perpetration of the crime. When the drunkenness has been the result of accident or collusion then the responsibility, as in the case of our own law, is much diminished. Hallucinations and illusions are a very common effect of drunkenness; and when these lead to the commission of any criminal act, then an exculpation, or at least some mitigation, ought to be admitted. (p. 610.) The author relates a remarkable case in point, where two friends being intoxicated, the one killed the other under an illusion that he was an evil spirit. The drunkenness of the accused was held to have been voluntary, and he was condemned to ten years' imprisonment with hard labour. (p. 635.) He thinks this sentence to have been too severe; but in another case which follows the prisoner was condemned to the galleys for life. A case of this description has been recently decided in our own country (March, 1840).* A man while intoxicated killed his friend, who was also intoxicated, under the illusion that he was some other person who had come to attack him. The judge made the prisoner's guilt to rest upon the fact whether had he been sober he would have perpetrated the act under a similar illusion? As he had voluntarily brought himself into a state of intoxication that was no justification. He was found guilty of manslaughter, and sentenced to two months' imprisonment.

The excitement produced by narcotics very much resembles that of intoxication; and here in the author's view the question of responsibility for acts committed during this state should be decided by the fact whether the drug had been voluntarily taken, or collusively or accidentally administered. (p. 650.) This question may seriously affect those who are addicted to opium eating, and of whom the number in this country is said to be annually increasing. The rule of the English law is, that when an act is perpetrated by an individual labouring under a frenzy excited by drugs, administered medicinally or designedly by another, he is no longer responsible for it.

M. Marc's views relative to the responsibility of persons waking from sleep or in a state of somnambulism are similar to those which have been given in a former part of this article. We must, he considers, be mainly guided by the fact, whether there may have existed any interest or motive on the part of the accused for the perpetration of the crime.

The work closes with an account of the civil jurisprudence of insanity, to which, comparatively speaking, but a very small space is devoted. In respect to the validity of wills and deeds, the French law is very simple. "In order that a deed should be valid, the individual making it must be of sound mind." (Art. 901, Code Civile.) The author contents himself with illustrating this subject by cases. He recommends medical witnesses to be careful how they trust to the statements of interested parties, or found their opinions thereon. The first case is one of some interest. We learn by it that, in deciding on the validity of wills, the French law looks to all the acts of the party, and his general habits; much reliance is also placed on the manner in which the deed is expressed, if drawn up by the testator himself." Suicide following the making of a will is not

* Norfolk Circuit, Aylesbury. Patteson's case, March 10, 1840.

to be taken as a proof of insanity, without reference to other circumstances. The individual in the above instance destroyed himself six days after its execution; but the will was not invalidated on this account. By a case decided in our own courts, we find that a will was not declared invalid, although the instructions for it had been given only three days before the testator committed suicide.

A man may be affected with any bodily disease; but unless the mind be directly or indirectly disturbed, his civil acts are valid for all legal purposes. A will was contested on the ground that the testator laboured under hemiplegia while executing it, but it was properly represented by Esquirol that, although hemiplegia may affect the brain, a circumstance indicated by the sight, hearing, and other senses becoming weakened, yet this does not necessarily indicate an impairment of the understanding. A man's mind under these circumstances may not be so strong as in robust health, but still it may retain a disposing power.

In wills made by the insane, we have seen that the proof of a lucid interval is often a matter as important as it is difficult. If the person have been already declared insane, the proof of the lucid interval rests with the devisee, and this must not depend upon probability, but upon certainty. If the person has been proved insane, then after the proof of general insanity, the existence of a lucid interval during the act must be established by those who would benefit by it. The following observations of M. Marc are good:

"Idiocy and imbecility are never accompanied by lucid intervals. In dementia, they are only met with in those cases where the dementia is not chronic, or has directly succeeded a state of intermittent or periodical mania; and then it is characterized rather by a sort of stupor or moral annihilation, (anéantissement moral) than by an incoherence of ideas. Among the different forms of insanity, mania is that state in which lucid intervals, such as the law demands for the validity of acts, are the most frequent and perfect." (*Marc*, p. 704.)

From the remarks on "Interdiction," we learn that the cumbrous machinery of lunacy commissions does not exist in France, and that a man's property may be there legally protected without a large part of it being spent to determine whether or not he should have the control of it. The French law appears to us remarkably clear on this subject. By Article 489, Code Civile, we learn that "where an adult person labours under habitual imbecility, dementia, or mania, whether with or without lucid intervals, he is a fit subject for interdiction." Then again, for all the cases which this provision does not cover, he may, if discharged from interdiction, have a committee appointed, without whose consent no civil act of his, will be valid. (Article 499, Code Civile, p. 706.) This last article would provide for numerous cases of mental weakness with incompetency, in which the verdicts of English commissioners are so uncertain and vacillating as to allow of no general rule being drawn. A principle of this kind prevails in the law of Scotland; but in England nothing of the sort exists; and hence every contested case becomes a battle in which legal ingenuity is often triumphant. According to the French system medical opinions are not commonly required where the mere appointment of a provisional committee is in question. The general habits of life, conduct, and a personal examination of the party are the points which govern the decision of the court. In matters of difficulty, however, physicians are very properly consulted.

The admissibility of the insane as witnesses in courts of law, M. Marc thinks, is rather a question for the court than for a medical practitioner. A lunatic in this respect, may be compared to a witness incompetent from age, and hence it is impossible to lay down a general rule. Much depends on the form and degree of the mental affection. "Those who labour under an extreme degree of imbecility, mania, or dementia with defective memory, are not fitted to give evidence." (p. 716.) In England, where in any case the evidence of a witness confined in a lunatic asylum has been tendered, it has been usual to infer his insanity and declare his incompetency as such, until good proof is afforded of his having had a lucid interval at or about the time of the occurrence to which he testifies.

But little is said on the application of *restraint* and the laws regulating the confinement of lunatics in public and private asylums, the author considering that this subject has been already exhausted by Esquirol. (p. 716.) In England the statute 2 & 3 W. IV. c. 107, ss. 27 & 28, requires that the certificate to confine a lunatic should be signed by two medical practitioners, not in partnership nor having any interest in the asylum, who must have separately visited and personally examined the patient to whom it relates, not more than *seven clear days* previous to such confinement. We make this statement because we are certain that many practitioners are in the habit of signing certificates without being aware of the facility with which they may subject themselves to a trial for misdemeanour. According to the French law, issued July 6, 1838, the certificate of confinement must be signed by one physician within fifteen days of the time of its presentation. The physician must not belong to the asylum, nor be related to the lunatic, to the proprietors of the asylum, or the person demanding the confinement. The keepers of asylums are placed by this law under very strict jurisdiction, indeed so strict, that Esquirol considers some of the provisions for this reason inoperative. A full account of this law is given in the Ann. d'Hygiène, (vol. xxii. p. 215, 1839.)

The author's observations on the responsibility of the deaf and dumb, correspond to those which have been already made elsewhere. The volume is closed with the excellent remark, that "whether in civil or criminal law, there is a close connexion between the rational principles derived from the physical and moral study of man; and those which form the foundation of a sound theory of jurisprudence."

We conclude our notice with the expression of our opinion that M. Marc has treated this subject in a masterly manner. We see throughout the hand of the physician, the medical jurist, and the philanthropist; and much do we regret that the tomb has now finally closed over the labours of this able and industrious writer. The work reflects much credit on French medical literature, and it will, we think, be highly valued by the medical jurists of Germany, America, and England.

